# Williams Syndrome and Music: A Systematic Integrative Review

**DOI:** 10.3389/fpsyg.2018.02203

**Published:** 2018-11-14

**Authors:** Donovon Thakur, Marilee A. Martens, David S. Smith, Ed Roth

**Affiliations:** ^1^Department of Music Therapy, School of Music, Western Michigan University, Kalamazoo, MI, United States; ^2^Department of Behavioral Health, Child Development Center, Nationwide Children's Hospital, Columbus, OH, United States; ^3^Department of Pediatrics, College of Medicine, The Ohio State University Wexner Medical Center, Columbus, OH, United States; ^4^Brain Research and Interdisciplinary Neurosciences Laboratory, School of Music, Western Michigan University, Kalamazoo, MI, United States

**Keywords:** Williams syndrome, review, musicality, affect, auditory processing, imaging, cognition, anxiety

## Abstract

**Background:** Researchers and clinicians have often cited a strong relationship between individuals with Williams syndrome (WS) and music. This review systematically identified, analyzed, and synthesized research findings related to WS and music.

**Methods:** Thirty-one articles were identified that examined this relationship and were divided into seven areas. This process covered a diverse array of methodologies, with aims to: (1) report current findings; (2) assess methodological quality; and (3) discuss the potential implications and considerations for the clinical use of music with this population.

**Results:** Results indicate that individuals with WS demonstrate a high degree of variability in skill and engagement in music, presenting with musical skills that are more in line with their cognitive abilities than chronological age (CA). Musical strengths for this population appear to be based more in musicality and expressivity than formal musical skills, which are expressed through a heightened interest in music, a greater propensity toward musical activities, and a heightened emotional responsiveness to music. Individuals with WS seem to conserve the overall structure of musical phrases better than they can discriminate or reproduce them exactly. The affinity for music often found in this population may be rooted in atypical auditory processing, autonomic irregularities, and differential neurobiology.

**Conclusions:** More studies are needed to explore how this affinity for music can be harnessed in clinical and educational interventions.

## Introduction

### Rationale

The earliest accounts of Williams syndrome (WS) were identified in descriptions of case studies with two seemingly unrelated sets of characteristics (see Berdon et al., 2011—for review). In the 1950s, cases were reported of infantile hypercalcemia, failure to thrive, a distinctive facial appearance, and developmental delays (Schlesinger et al., 1956; Bongiovanni et al., 1957). In the early 1960s, cases identified by New Zealand cardiologist John C. P. Williams and German physician Alois Beuren were characterized by supravalvular aortic stenosis (SVAS) along with a similar presentation of persistent growth failure, distinctive facial appearances, developmental delays, and an overly-friendly personality (Williams et al., 1961; Beuren et al., 1962). By the mid-1960s, case reports describing individuals with both hypercalcemia and SVAS, along with other features common to both phenotypes, suggested that these seemingly unrelated presentations were variations of the same phenotype (Berdon et al., 2011).

WS (also known as Williams-Beuren syndrome) is a rare neurodevelopmental disorder caused by a hemizygous microdeletion on chromosome 7 (7q11.23). The deletion is composed of approximately 26-28 genes and commonly includes one allele of the elastin gene, with an estimated prevalence of 1 in 7,500–10,000 live births (Ewart et al., 1993; Strømme et al., 2002). Contemporary methods of diagnosis include laboratory tests such as *fluorescence in situ hybridization* (FISH) and array comparative genomic hybridization (Pober, 2010).

Individuals with WS commonly present with a unique array of medical, developmental, and social-emotional qualities. Distinctive facial features include: almond-shaped eyes, a stellate pattern in the iris, high and prominent cheekbones, a flat nasal bridge with upturned nose, full lips, a broad mouth, and abnormal dentition (Levitin and Bellugi, 1998). Medically, individuals with WS may experience cardiovascular abnormalities, hypertension, hypercalcemia, precocious puberty, low muscle tone, and curvatures of the spine (Pober, 2010). A higher incidence of hearing sensitivities has also been reported, including: hyperacusis (lowered threshold and higher detectability for hearing sounds), odynacusis (lowered pain threshold for sound volume), auditory aversions (fear of certain types of sounds at normal volume), and auditory fascinations (strong attraction to certain types of sounds) (Levitin et al., 2005).

Individuals with WS have been anecdotally described as unusually social, friendly, polite, highly empathetic, irresistibly drawn to strangers, and driven by social interaction (Doyle et al., 2004; Plesa Skwerer and Tager-Flusberg, 2016). Compared to peers, individuals with WS are described as more hyperactive (~65% meet DSM-IV criteria for ADHD), distractible, anxious, and more prone to developing specific phobias (Dykens, 2003; Leyfer et al., 2006). Järvinen et al. (2012) remarked on “intriguing dissociations” apparent in WS, which include: overly friendly behavior with difficulty making friends, social fearlessness coupled with anxiety, and abundant positive affect with maladaptive behaviors. Individuals with WS fall in the mild to moderate range of intellectual disability, with mean IQ scores falling between 50 and 60, a range of 40–100, and IQ scores remaining relatively stable with age (Martens et al., 2008). Many individuals with WS struggle with deficits in global vs. local processing of visual stimuli (Bihrle et al., 1989), visuospatial skills (Farran, 2005), and pragmatics (Brock, 2007). However, these individuals also possess relative strengths in expressive language, receptive vocabulary, phonological memory, facial perception and processing, and music (Brock, 2007; Martens et al., 2008).

The relationship between individuals with WS and music has been recognized even from early case reports. These reports described children with WS as having good singing skills (von Arnim and Engel, 1964) and an ability to learn songs with ease (Udwin et al., 1987). Individuals with WS demonstrate a high engagement in musical activities, express an interest in music at an early age, and exhibit a heightened emotional responsiveness to music (Don et al., 1999; Levitin et al., 2004; Dykens et al., 2005).

Early investigation into the musical skills of individuals with WS remarked on apparently enhanced or preserved abilities in music, including: a keen sense of pitch in reproducing songs, an enhanced skill for producing rhythms, and greater musical creativity (Lenhoff et al., 1997; Levitin and Bellugi, 1998). Later studies have yielded more precise and mixed results, suggesting that musical abilities in individuals with WS are more likely to be areas of relative strength rather than preserved functioning (Hopyan et al., 2001; Martínez-Castilla and Sotillo, 2008; Martens et al., 2010; Martínez-Castilla et al., 2011).

The curious relationship between individuals with WS and music has been the subject of multiple articles and empirical studies over the past 20 years. Currently, no formal review has been conducted to examine the relationship between WS and music through a comprehensive research lens. Notably, there are also no published articles examining the clinical use of music with individuals with WS. Collecting and analyzing available research on the relationship between WS and music could guide future inquiry in all areas and serve as a platform for future research on the clinical potential for music with this population.

### Objectives

The purpose of this review was to systematically identify, analyze, and synthesize research findings related to WS and music. This process covered a diverse array of methodologies, aiming to: (1) report current findings; (2) assess methodological quality; and (3) discuss the potential implications and considerations for the clinical use of music with this population.

## Methods

### Study design

This review borrowed from methodologies of systematic and integrative reviews.

A *systematic review* is a methodologically rigorous and comprehensive review of literature intended to identify, select, and critically appraise relevant research. A systematic review attempts to answer a specific research question by gathering and analyzing all empirical evidence that meets predetermined criteria. It follows explicit and systematic methods to reduce bias and enhance the quality of the findings. The studies included in these reviews tend to follow similar methodologies and/or report similar outcomes (Moher et al., 2009; The Cochrane Collaboration, 2011).

An *integrative review* is a broader approach that includes studies which utilize different methodologies, including both theoretical and empirical literature, in order to gain a greater perspective of a particular phenomenon. Integrative reviews incorporate a wide range of purposes: to define concepts, to review theories, to review evidence, and to analyze methodological issues of a particular topic. However, analyzing and synthesizing varied primary sources is a major challenge in undertaking an integrative review and leaves room for bias and error. The studies included in the integrative review often need to be divided into subgroups according to some logical system to facilitate analysis (Whittemore and Knafl, 2005; Souza et al., 2010).

This review follows the framework of an integrative review with respect to the inclusion and analysis of research from a diverse set of methodologies and covers a range of topics related to WS and music. In order to enhance the rigor and reduce the risk for bias or error, the formal search for literature more closely mirrored that of a systematic review in an attempt to identify, select, and compile a comprehensive database of primary sources related to this topic.

### Search strategy

An electronic search was conducted from the following databases: PubMed, PubMed Central, EMBASE, SCOPUS, Web of Science, EBSCO, and ProQuest. These databases were searched using the terms: “Williams syndrome” and “music.” Additionally, reference lists from included articles were reviewed for other relevant articles.

The following inclusion and exclusion criteria were used to determine eligibility.

Criteria for Inclusion:

- Date Range: published before 2017 (through December 2016)
- Subject: Williams syndrome
- Subject: music
- Participants (where applicable): Williams syndrome
- Published in English
- Published in a peer-reviewed journal

Criteria for Exclusion:

- Review article.

### Data analysis

Search results were managed using Endnote X8. After all database searches were complete and duplicates removed, all remaining search results were first screened for inclusion based on the title and abstract. Finally, the remaining articles were screened using the full-text of the article (For a full list of exclusions based on full-text, see Appendix A in Supplementary Material). All screening was duplicated by a secondary independent reviewer trained in following the aforementioned criteria in order to prevent the omission of potentially relevant articles. All discrepancies were settled between the two reviewers.

The primary author extracted data from the included articles using a Data Extraction Form created for this study (Appendix B in Supplementary Material). Two secondary independent reviewers, trained in the use of the extraction form, each reviewed a random selection of 25% of the articles using sections: 4: Participants and Groups; 5: Tasks and Outcomes; and 7: Other Information. Findings from each of the secondary reviewers were reviewed with the primary author; all discrepancies were settled between the two reviewers. Data extracted by the reviewers were collated and integrated into summary tables and a narrative synthesis depicting their notable findings, converging results, and methodological limitations.

## Results

### Study selection and characteristics

**Figure 1:**
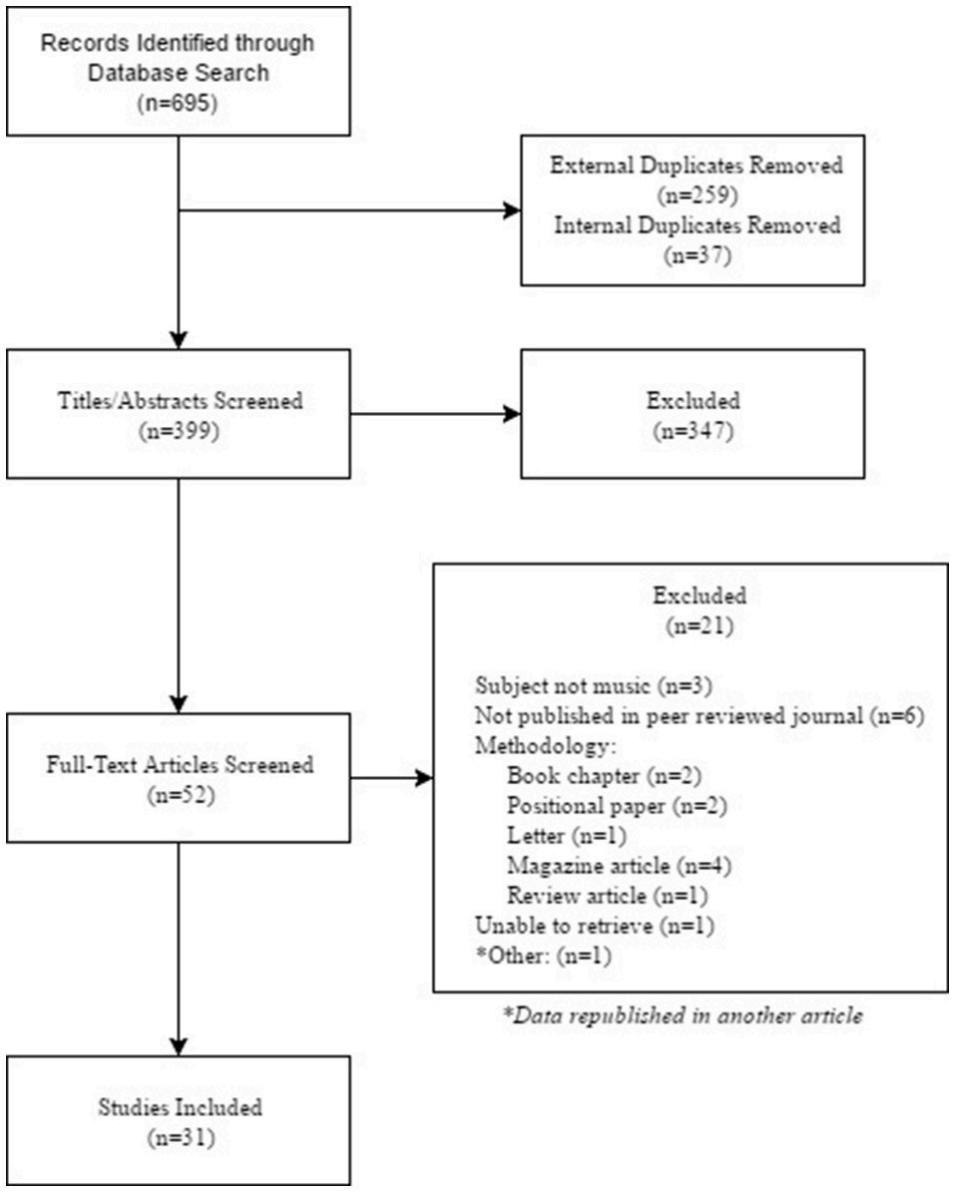
PRISMA flow diagram.

The formal search identified 31 articles that met the criteria for inclusion (Figure 1). These articles were allocated into seven groups based similarities in their outcomes: Musicality, Musical Skill, Emotional Responsiveness, Musical Processing, Brain Imaging and Morphology, Cognitive Processes, and Fears, Anxieties, and Problem Behaviors (Table 1). Some of the articles were included in multiple groups. Areas where findings related to one of these sections appear outside of that section are often when they were explained as a co-variate or impacted the results in relation to that section. Overall, the 31 articles included 38 studies as some of the articles included more than one study. Two of these articles (Järvinen et al., 2012; Lense and Dykens, 2013b) included only one study that met the criteria for inclusion. Only data from that study was extracted for analysis. Of note, 100% of the included articles were indexed within the ProQuest database.

**Table 1:** Number of articles included in each area.

**Topic**	Number of articles^*^
Musicality: affinity, experience, and engagement	10
Musical skill: tonal and rhythmic skills	9
Emotional responsiveness	12
Musical processing: absolute pitch, amusia, and auditory processing	4
Brain imaging and morphology	5
Cognitive processes: memory and math	3
Fears, anxieties, and problem behaviors	3

* *Nine articles were included in two topic areas and three articles were included in three topic areas*.

### Synthesized findings

#### Musicality: affinity, experience, and engagement

Ten of the included articles reported findings related to musicality (Table 2). The attraction to music and overall musicality among individuals with WS has been anecdotally cited since early case reports (von Arnim and Engel, 1964; Udwin et al., 1987). Yet, the concept of “musicality” is difficult to define or quantify. Levitin (2012) recognized that musicality is quite heterogeneous and advocates for including a wide range of musical behaviors in the description of musicality. For the purposes of this paper, affinity (e.g., interest, preferences, enjoyment, motivation); experience [e.g., musical training, community involvement (i.e., band, choir, church, etc.)]; and engagement (e.g., time spent playing/listening, attending concerts) were included as aspects of musicality, apart from musical skill. Other facets may include creativity, expressivity, and sensitivity, which fall under the category of “engagement” in this paper (for findings related to emotional responsiveness, see Emotional Responsiveness section). Given the lack of formalized assessment measures in these areas, most of the studies included in this section utilized parent report via various questionnaires to examine various aspects of musicality. The most utilized tools among the included studies were the Musicality Interest Scale (MIS; Blomberg et al., 2006) and the Salk/McGill Music Inventory (SAMMI; Levitin et al., 2004), which define musicality as interest/liking in music, emotional reactions to music, and musical abilities. Many other studies in this paper utilized questionnaires to examine musicality indirectly, however did not report findings as a focus of their study (Blomberg et al., 2006; Dai et al., 2012; Lense and Dykens, 2013a; Lense et al., 2013, 2014b).

**Table 2:** Musicality: affinity, experience, and engagement.

**Author/Year**	***N***	**Age^a^ (years)**	**Task(s)**	**Finding(s)**
Dunning et al., 2015	(WS) 44	(WS) 8–48	Musicality questionnaire	Increased enjoyment and frequency of music listening compared to peers and siblings
Lense and Dykens, 2013b (study #2 only)	(WS) 13	(WS) 29.47 ± 6.35	Prepared solo music performance in front of mixed familiar/unfamiliar audience	Greater musical skill associated with starting lessons at younger age, exposure to multiple types of lessons, and amount of time currently spent playing music
Lense and Dykens, 2013a	(WS) 47	(WS) 7–49	Single semi-structured lesson on a novel instrument (Appalachian dulcimer); Musicality Interest Survey (MIS)^b^	Self-reported use of auditory learning strategies predicted greater skill on a novel instrument beyond previous musical skill and visual-motor integration *(provides considerations for musical education)*
Lense et al., 2013	(WS) 73	(WS) 10–51	Musicality Interest Survey (MIS)^b^ Amusia battery	High percentage of WS involved in musical training; Exposure to various types of musical training was a better predictor of musical skill than cumulative duration
Ng et al., 2013	(WS) 55 (TD) 19	(WS) 16–52 (TD) 18–41	Compared measures of musicality, sociability, and language comprehension; Salk McGill Music Inventory (SAMMI)^c^	Greater interest associated with greater linguistic capacity
Martens et al., 2011 (2 studies)	(WS) 38	(WS) 6–59	Musicality questionnaire	Increased enjoyment and frequency of music listening compared to peers and sibling
	(WS) 38	(WS) 7–50		
Dykens et al., 2005 (2 studies)	(WS) 31 (PW) 26 (DS) 32	(WS) 10.22 (4.86) (PW) 10.26 (4.86) (DS) 11.5 (4.49) (All) Range 4–21	Musicality questionnaire	WS more likely to take music lessons, play an instrument, and have higher ratings of musical skill
	(WS) 26 (PW) 16 (DS) 25	(WS) 20.88 (11.48) (PW) 19.38 (6.70) (DS) 18.83 (7.11) (All) Range 8–47		
Levitin et al., 2004	(WS) 118 (TD) 118 (ASD) 30 (DS) 40	(WS) 20.4 (10.4) Range: 5–50 (TD) 20.9 (7.4) Range: 5–44 (ASD) 18.2 (7.7) Range: 9–39 (DS) 17.2 (9.2) Range: 5–51	Salk McGill Music Inventory (SAMMI)^c^	Greater emotional responses to music, manifest interest in music at an earlier age, more hours per week listening to music than all other groups; Higher musical accomplishment, engagement, and interest than ASD/DS Seven musical factors predict group membership
Reis et al., 2003	(WS) 16	Unclear Only DOB given	10-day intensive music program	Use of a talent development approach improved musical skill and engagement *(provides considerations for musical education)*
Don et al., 1999	(WS) 19 (TD) 19	(WS) 8–13 (TD) 5–12	Parent interview and questionnaire	Greater interest and range of emotional responses to music *(provides considerations for musical education)*

*ASD, Autism Spectrum Disorder; DS, Down Syndrome; PW, Prader-Willi Syndrome; TD, Typically Developing Control; WS, Williams Syndrome*.

a *Age in years, expressed in one of the following formats based on information available: (a) range in years, (b) mean (standard deviation), or (c) mean ± years*.

b *Musicality Interest Scale (MIS): Questionnaire with subscales in: (1) interest in and liking of music, (2) emotional reactions to music, and (3) musical skills*.

c *Salk McGill Music Inventory (SAMMI): Comprehensive questionnaire with subscales in: (1) demographic information, (2) interest in music, (3) emotional responsiveness to music, (4) music creativity and reproduction, (5) musical training, (6) age of onset of musical behavior*.

Studies in this section continue to shed light on the WS phenotype with an overall high level of agreement. Levitin et al. (2004) conducted a comprehensive survey with the largest sample of individuals with WS included in this study (*n* = 118), as well as typically developing (TD) controls and two comparison groups of individuals with other neurodevelopmental disorders, Autism Spectrum Disorder (ASD) and Down Syndrome (DS). In comparison to all groups, individuals with WS manifest interest in music at an earlier age, spend more hours per week listening to music, and demonstrate higher and longer-lasting emotional responses to music (also Don et al., 1999).

Levitin et al. (2004) further report that, compared to TD peers and those with other neurodevelopmental disorders, individuals with WS play instruments more often and show a higher interest in music-related activities, but show similar frequency of spontaneously generating music (also Levitin and Bellugi, 1998; Don et al., 1999). When compared to only those with neurodevelopmental disorders, individuals with WS play or create original music and rhythms more frequently, are more accurate in their reproductions of songs, and are rated higher in musical skill and achievement (also Dykens et al., 2005). Martens et al. (2011) noted that nearly 50% of individuals with WS enjoy music significantly more than their siblings. Also, individuals with WS who demonstrate greater linguistic capacity tend to be more interested in music (Ng et al., 2013).

Between 50 and 90% of individuals with WS engage in some type of musical training (involvement in music lessons or participation in band/choir, Martens et al., 2011; Lense et al., 2013; Dunning et al., 2015). However, information about the musical training of participants is inconsistently reported across studies and may be subject to bias when participants are recruited from music camps. Many individuals with WS elect to participate in choir or band throughout school and are most likely to take lessons in piano, voice, or drums (Martens et al., 2011; Lense et al., 2013; Dunning et al., 2015).

Lense and Dykens (2013a) taught 46 children and adults with WS to play a novel instrument, the Appalachian dulcimer, in a 35min, semi-structured, adaptive lesson. Gained skill on the novel instrument was associated with the number of types of formal lessons, number of instruments played formally and informally, hours per day playing instruments or singing, parent report of musical skill, and better visual-motor integration ability. However, self-reported use of auditory learning strategies predicted greater skill on the dulcimer beyond musical skill (as rated by a solo musical performance) and visual-motor integration. Similarly, Lense and Dykens (2013b) reported that greater musical skill on a prepared solo music performance, as judged by trained judges, was associated with starting lessons at a younger age, exposure to multiple types of lessons, and amount of time currently spent playing music.

These studies suggest that the number of types of musical training, and not duration of individual lessons, may be a more reliable indicator of musical achievement in individuals with WS (see also Reis et al., 2003). Lense et al. (2013) suggest that this may be related to the fact that musical training for individuals with WS is often different and more inconsistent than with TD populations. Families of individuals with WS often experience difficulty in finding suitable teachers and individuals with WS may experience discouragement related to the fine motor skills required to play certain instruments. These families may be forced to seek out various teachers or multiple avenues for musical enrichment, with the only constant being the continual involvement in some type of musical activity.

Many of the included studies offered various considerations for the musical education of individuals with WS. To promote participant success, Lense and Dykens (2013a) selected an instrument that was challenging but didn't require intensive fine motor skills, placed numbered stickers along the frets, did not use written music—instead focused on repeated rhythms and patterns, and utilized a curriculum with a mixture of familiar song and improvisation. Don et al. (1999) discussed adaptations concerning physical and cognitive limitations and suggested that training could incorporate shorter sessions with a predictable routine, include simple tasks such as imitation and repetition, and provide opportunities for creative and emotional expression. Reis et al. (2003) also point out a preference for social, auditory, and group learning in individuals with WS. Taken together, these considerations suggest that musical training for individuals with WS may be better suited toward a focus on strengths, learning preferences, and the development of musical expressiveness through play and improvisation, as opposed to learning specific skills such as reading music and learning scales or notes (Hopyan et al., 2001).

Lense et al. (2013) is the only included study to directly examine musical training, which was evaluated by implementing a single semi-structured lesson. Longitudinal studies of musical development and studies examining the effects of repeated musical training over time would help to increase understanding of learning strategies and supports necessary for individuals with WS. Future studies are needed to examine the impact of these proposed supports on musical training and may have implications for other educational or clinical interventions.

Overall, the long-cited claims of an “affinity” for music in individuals with WS appear to be expressed through an earlier and higher interest in music or music-related activities, more time spent listening to or playing music, and a natural sense of musical creativity. These findings may be relevant to future educational or clinical study as they may translate to increased motivation for intervention.

#### Musical skill: tonal and rhythmic skills

Nine articles assessed a wide variety of tonal and rhythmic skills (Table 3). Given the complexities in measuring these skills and their component parts, these articles utilized a wide variety of tasks and outcome tools, with very little overlap in standardized measures. However, assessment tasks used within these studies tended to follow similar structures.

**Table 3:** Musical skill: tonal and rhythmic skills.

**Author/Year**	***N***	**Age^a^ (years)**	**Skill(s)**	**Task(s)/Tool(s)**	**Finding(s)**
Lense and Dykens, 2016 (2 studies)	(WS) 74 (TD) 52	(WS) 26.4 ± 9.6 (TD) 24.3 ± 9.4	Beat perception Meter perception	Beat Alignment Test (BAT)^b^: determine if a metronome matched the beat of a musical passage; Montreal Battery of Evaluation of Amusia-meter subtest (MBEA-m)^c^: determine if a melody was in duple or triple meter	WS < TD CA on both beat and meter perception; High degree of individual variability in both groups
	(WS) 50	(WS-BAT^b^) 26.2 ± 8.4 (WS-MBEA^c^) 26.8 ± 8.3	Beat perception Meter perception	Compared measures of beat and meter perception to measures of cognitive and adaptive function (Vineland-II)	Beat perception significantly associated with communication and socialization Meter perception significantly associated with socialization
Martínez-Castilla et al., 2016	(WS) 20 (TD) 54	(WS) 6–17 (TD) 4–17	Pitch discrimination Chord discrimination Dissonance perception Tonal closure	Compared performance pitch related skills to CA and standardized measures of cognitive development	WS showed atypical development of pitch-related skills
Martínez-Castilla and Sotillo, 2014	(WS) 14 (TD) 26	(WS) 8–17 (TD) 8–17	Pitch discrimination	Compared pitch processing in both music and prosody discrimination	WS < TD CA on pitch and prosody discrimination; Pitch discrimination in music predicted pitch discrimination in prosody
Martínez-Castilla et al., 2011	(WS) 20 (TD) 30	(WS) 20.10 (5.87) Range: 12–32 (TD) 20.03 (6.20)	Rhythm discrimination Rhythm production	Same/different discrimination task Echo clapping task	WS < TD CA on rhythm discrimination and production; Rhythm skills were affected by IQ
Martens et al., 2010	(WS) 25 (TD) 25	(WS) 8–41 (TD) 8–41	Various tonal and rhythm skills in discrimination and production	Specimen Aural Test (SAT)^d^ Bentley Measures of Musical Abilities^e^	WS < TD CA on tonal and rhythmic perceptual and production tasks; WS = TD clapping in time to beat of musical passage
Martínez-Castilla and Sotillo, 2008 (2 studies)	(WS) 7 (TD) 7	(WS) 10–30 (TD) 10–30	Singing	Singing a familiar song, measured using both acoustical analysis and perceptual judgments by musicians/non-musicians	WS < TD CA singing skill; Those with musical training performed better;
	(WS) 15 (TD) 15	(WS) 17–32 (TD) 17–32	Pitch matching Singing	Pitch matching task; Singing a familiar song, measured using both acoustical analysis and perceptual judgments by musicians/non-musicians	WS may benefit less from musical training than D
Hopyan et al., 2001	(WS) 14 (TD) 14	(WS) 12 (3) (TD) 12 (3)	Pitch discrimination Rhythm discrimination Melodic imagery Phrasing perception	Primary Measures of Music Audiation (PMMA)^f^; Musical Aptitude Profile (MAP)^g^	WS < TD CA on pitch and rhythm discrimination; WS = TD CA on perception of musical expressiveness
Don et al., 1999	(WS) 19 (TD) 19	(WS) 8–13 (TD) 5–12	Pitch discrimination Rhythm discrimination	Primary Measures of Music Audiation (PMMA)^f^	WS = TD MA on pitch discrimination WS < TD MA on rhythm discrimination; Music and language skills moderately correlated
Levitin and Bellugi, 1998	(WS) 8 (TD) 8	(WS) 9–20 (TD) 5–7	Rhythm production	Echo clapping task	WS = TD MA

*CA, Chronological Age; MA, Mental Age; TD, Typically Developing Control; WS, Williams Syndrome*.

a *Age in years, expressed in one of the following formats based on information available: (a) range in years, (b) mean (standard deviation), or (c) mean ± years*.

b *BAT, Beat Alignment Test (Iversen and Patel, 2008)*.

c *MBEA-m, Montreal Battery of Evaluation of Amusia-meter subtest (Peretz et al., 2003)*.

d *SAT, Specimen Aural Test (Nickson and Black, 1962)*.

e *Bentley, Bentley Measures of Musical Abilities (Bentley, 1985)*.

f *PMMA, Primary Measures of Music Audiation (Gordon, 1986)*.

g *MAP, Musical Aptitude Profile (Gordon, 1995)*.

Earlier studies reported similar performance on tonal and rhythmic tasks when compared to peers matched for mental age (MA) (pitch discrimination: Don et al., 1999; rhythm production: Levitin and Bellugi, 1998). However, when compared to TD peers matched for chronological age (CA), subsequent studies more consistently reported that individuals with WS tend to perform below their TD peers on both tonal and rhythm skills (pitch discrimination: Hopyan et al., 2001; Martens et al., 2010; Martínez-Castilla and Sotillo, 2014; rhythm discrimination: Hopyan et al., 2001; Martens et al., 2010; Martínez-Castilla et al., 2011; pitch production/singing: Martínez-Castilla and Sotillo, 2008; Martens et al., 2010; rhythm production: Martens et al., 2010; Martínez-Castilla et al., 2011; beat and meter perception: Lense and Dykens, 2016) and equivalent to TD peers on perception of musical expressiveness (Hopyan et al., 2001).

##### Tonal skills

The tonal skills assessed in these studies encompass a variety of skills such as being able to match pitches, sing melodies accurately, distinguish if pairs of notes, chords, or melodies are the same or different, identify the number of notes in a chord, sing the final note of a melodic phrase, perceive dissonance, and detect errors in phrasing and melodic contour (Claims about the prevalence of absolute pitch (AP) in WS have yielded mixed results and are discussed in a later section, see Absolute Pitch section).

Martínez-Castilla and Sotillo (2008) utilized both acoustical analysis and perceptual judgments from both musicians and non-musicians to assess singing skills by having participants sing two well-known songs. Individuals with WS were determined to have significantly worse singing skills than TD individuals matched for CA. Acoustical analysis revealed that individuals with WS produced significantly more tuning and interval errors (e.g., a wrong pitch), marginally more contour errors (e.g., a pitch error that falls in the opposite direction than that of the target pitch, changing the overall contour of the melody), and had poorer key stability. Participants with musical training in both groups tended to sing at a slower tempo, which is likely to have reduced pitch errors. However, participants with WS also made more time errors overall, independent of musical training. Perceptual judgments by musicians rated individuals with WS as having worse intonation, yet those with musical training were perceived to have improved intonation over those without musical training. These findings appear to highlight the benefits of musical training. However, despite poorer performance, the musically trained individuals with WS in this study had more training than the TD controls, which might suggest that they benefit from training to a lesser degree than TD peers or that individuals with WS may need more training to reach similar levels of accomplishment (Lense and Dykens, 2013a; Lense et al., 2013; and Martens et al., 2010 also share effects of musical training).

Martínez-Castilla et al. (2016) examined and compared the development of pitch-related skills in individuals with WS and TD peers matched for CA. Performance on four pitch-related tasks was compared to CA and a battery of cognitive skills. Findings revealed that the development of pitch-related skills is atypical in WS. For the TD group, performance on all tasks improved with CA and higher performance on standardized measures of cognitive development. The TD group also demonstrated a linear developmental progression of skills: pitch discrimination developed first, chord discrimination and dissonance perception developed later, followed by tonal closure. This progression is logical in that the discrimination of pitch involves processing of individual notes; discrimination of chords and perception of dissonance involves processing of multiple notes and early harmonic structure; and finally, tonal closure involves decisions based on established tonal relationships (i.e., individual pitches combine to form chords and melodies, which combine to establish key and tonality).

Individuals with WS demonstrated a less clear pattern: development of pitch discrimination preceded chord discrimination and tonal closure, yet no other developmental relationships were found, despite performance above chance on all tasks. For the individuals with WS, CA predicted chord discrimination; matrix reasoning predicted chord discrimination and tonal closure; and backward digit-span predicted pitch discrimination, chord discrimination, and tonal closure. These findings are also logical in that chord discrimination and tonal closure involve perception of cadential and harmonic relationships, similar to the skills necessary to perceive patterns in matrix reasoning. Also, the skills predicted by backward digit-span require auditory working memory to store and compare stimuli. Thus, although the atypical development of pitch-related skills in WS may not develop linearly or in synchrony, this may be a reflection of the atypical cognitive development in WS as the development of specific pitch skills was predicted by the requisite cognitive skills, namely matrix reasoning and backward digit-span.

Given the heightened language abilities of individuals with WS and that processing of both auditory language and music involves the processing of similar components (such as pitch, volume, and duration), two studies examined the relationship between musical skills and various language skills. Don et al. (1999) compared performance on a language skills battery with performance on standardized tests for tonal and rhythmic discrimination. All language measures were moderately correlated with both tonal and rhythmic musical skill. However, this was also true for the control group of TD peers matched by MA (based on receptive vocabulary), which may indicate a developmental relationship between these domains. Furthermore, Martínez-Castilla and Sotillo (2014) reported that the ability to discriminate musical pitches predicted performance on a pitch-based prosody task in WS, suggesting that pitch processing in music and prosody may share an underlying mechanism in WS.

Although tonal skills in WS appear to more consistently fall below that of TD peers of similar CA, this may be related to a variety of factors, such as: atypical development of pitch skills, general cognitive deficits, or differences in the benefits received from formal musical training. Future studies are needed to examine the extent to which these factors influence tonal skills in individuals with WS.

##### Rhythmic skills

The rhythm skills assessed in these studies also encompassed a variety of skills, such as being able to distinguish if pairs of rhythmic patterns are the same or different, repeating rhythmic patterns accurately, clapping in time to the beat of a musical passage, detecting changes in tempo over time, and maintaining stable tempo over a musical performance.

Earlier studies of rhythmic skills focused more on discrimination than production tasks and only assessed one of these skills at a time. Martínez-Castilla et al. (2011) presented the first study to assess both rhythmic production and discrimination skills in the same sample of adolescents and adults with WS. Given the discrepant results of previous studies, Martínez-Castilla and colleagues also sought to remediate methodological limitations of previous rhythm studies (Levitin and Bellugi, 1998; Don et al., 1999; Hopyan et al., 2001) related to sample size, heterogeneity of sample, cognitive measurement, matching of control groups, and recording artifacts. Overall, individuals with WS performed significantly worse than TD controls matched for CA on both the discrimination and production tasks. Rhythm skills were also affected by cognitive level, as the difference between WS and TD individuals lost significance when controlled for IQ. This stands in contrast to Don et al. (1999), who found that individuals with WS performed at a level below their MA on a rhythm discrimination task.

Lense and Dykens (2016) extended their assessment of rhythm skills beyond discrimination and production tasks, instead examining beat and meter perception skill. Consistent with other rhythm studies in WS, both beat and meter perception skills in participants with WS fell below that of TD controls matched for CA with similar levels of musical training, although the authors reported a high degree of individual variability in both groups. Performance on beat and meter perception tasks were correlated, which makes logical sense as the perception of beat is a precursor to perceiving meter. Both of these skills were also predicted by IQ and a tendency toward a fundamental-processing style in the WS group (see Auditory Processing section). Greater performance was associated with cumulative years and the number of different types of musical training.

Fewer studies have examined rhythm production skills than perceptual abilities for rhythm. Individuals with WS perform worse than CA-matched TD peers on tasks requiring them to repeat rhythmic patterns, either by singing or clapping (Martens et al., 2010; Martínez-Castilla et al., 2011). One study reported equivalent abilities in both WS and TD individuals in the ability to clap in time to the beat of a musical passage (Martens et al., 2010).

Qualitatively, Levitin and Bellugi (1998) reported a few observations following their study requiring participants to repeat various rhythmic patterns by clapping. First, individuals with WS and peers matched for MA demonstrated good temporal conservation. In other words, if a participant made an error by producing more or fewer notes than the target pattern, they also generally altered the length of the notes to match the overall length of the target. Second, when individuals with WS made an error in reproducing a rhythm, their errors were more likely to remain rhythmically consonant or compatible with the target rhythm. The authors viewed these errors as “creative completions” or extensions of the target pattern. This was not observed in the control group. However, Martínez-Castilla et al. (2011) reported the opposite relationship when employing a similar task using a TD control group matched for CA, whereby age-matched TD controls produced more creative completions than their WS counterparts. One explanation for this difference could be related to age, as participants with WS in Levitin and Bellugi's study were older/matched by MA, perhaps allowing time for more musical experiences, while in Martínez-Castilla et al.'s study the participants were matched for CA.

Similar to tonal skills, rhythm skills in WS also appear to more consistently fall below that of TD peers of similar CA. However, given the discrepant findings reported here, particularly within production skills, future studies assessing both perceptual and production skills for rhythm using the same sample are needed. Also, an examination of the development of rhythmic skills in individuals with WS, similar to the developmental trajectory approach used by Martínez-Castilla et al. (2016), is needed to continue to shed light on the relationship between musical skills and other cognitive processes.

Interestingly, many of the authors in this section also reported anecdotal findings related to the participants with WS in their studies. Tonally, Martínez-Castilla and Sotillo (2008) reported that participants with WS sang with more “decoration,” personality, expressiveness, and creativity. Rhythmically, Levitin and Bellugi (1998) reported that participants with WS demonstrated a strong sense of phrasing and meter, sensitivity to rhythmic changes, and musical creativity in rhythm (i.e., creative completions). Therefore, musical strengths in this population may fall more within their expressiveness or creativity in music than within specific formal skills. However, these observations may stand in contrast to behaviors of TD controls on the basis that individuals with WS may be less inhibited or more performative in a lab setting than TD peers.

Overall, individuals with WS do not appear to present with preserved function in musical skills. Specifically, they are aware of the structure of musical phrases, but aren't always able to discriminate or reproduce the phrases accurately. In addition, they appear to present with “relatively” good skills in relationship to their cognitive level. As these skills have been shown to be affected by cognitive profile, it will be important to include assessment of cognitive skills as part of studies examining musical skill. Also, considering this relationship, studies examining the impact of musical training on cognitive skills in WS are needed as this may be a motivating avenue to practice and acquire more complex cognitive skills. Further inquiry into the relationship between specific cognitive skills and specific musical skills, particularly from a developmental framework, could be important in interpreting the results of these and future studies.

An overall limitation of the studies included in this section is the lack of control groups with other developmental delays. Given the discrepant findings between articles related to matching for CA vs. MA and the relationships between musical skill and cognitive function, study of various musical skills in WS in comparison to other developmentally delayed populations may shed light on these relationships and the musical skills profile of individuals with WS.

Another consideration in assessing the musical skills of individuals is the presentation of the musical stimulus. Given the salience of both musical and social experiences, heightened engagement in musical activity, and desire to please found in this population, presenting stimuli live (i.e., by an individual) may create bias when compared to stimuli presented via recording. Future studies are needed to investigate the impact of stimulus type on musical behavior.

#### Emotional responsiveness

Twelve articles reported findings related to emotional responsiveness to music (Table 4). These findings were collected using a variety of measures, including: parent report via questionnaires and interviews, various affect identification tasks, and measures of autonomic reactivity to musical and emotional stimuli.

**Table 4:** Emotional responsiveness.

**Author/Year**	***N***	**Age^a^ (years)**	**Task(s)**	**Finding(s)**
Järvinen et al., 2016	(WS) 12 (ASD) 17 (TD) 20	(WS) 10–14 (ASD) 8–14 (TD) 8–13	Affect identification task (auditory)	WS = TD CA = ASD identifying auditory affect; WS social > non-social affect; WS increased arousal (HR variability and EDA) to vocalizations and music; WS diminished habituation to both vocalizations and music
Lense et al., 2014b	(WS) 13 (TD) 13	(WS) 27.1 ± 7.1 (TD) 27.7 ± 6.0	Affect identification task (visual) With auditory prime	WS = TD CA WS faster reaction time when visual and auditory stimuli congruent > incongruent
Lense et al., 2013	(WS) 73	(WS) 10–51	Musicality Interest Scale (MIS)^b^	Emotional responsiveness was predicted by auditory sensitivities
Ng et al., 2013	(WS) 55 (TD) 19	(WS) 16–52 (TD) 18–41	Compared measures of musicality, sociability, and language comprehension.	Significant correlation between emotional responsivity to music and social-emotionality
Dai et al., 2012	(WS) 13 (TD) 9	(WS) 19–42 (TD) 19–45	OT and AVP measured during music and cold pressor test	WS higher baseline OT; Increased OT and AVP in response to music and cold pressor
Järvinen et al., 2012(study #2 only)	(WS) 20 (TD) 26	(WS) 13–46 (TD) 18–31	Affect identification task (auditory)	WS < TD CA identifying auditory affect; WS social > non-social affect; WS increased arousal (HR variability) to vocal affect > music affect
Bhatara et al., 2010	(WS) 11 (ASD) 23 (TD) 23	(WS) 13–43 (ASD) 11–20 (TD) 13–16	Affect identification task (auditory-expressivity in musical performance)	WS = TD CA; WS > ASD Recognizing emotion in musical performance
Järvinen-Pasley et al., 2010	(WS) 21 (TD) 21 (DD) 16	(WS) 12–40 (TD) 12–39 (DD) 18–52	Affect identification task (visual) With musical prime	WS = TD CA (social) WS < TD CA/WS > DD WS social > non-social affect; No difference when visual and auditory stimuli congruent > incongruent
Dykens et al., 2005(2 studies)	(WS) 31 (PW) 26 (DS) 32	(WS) 10.22 (4.86) (PW) 10.26 (4.86) (DS) 11.5 (4.49) (All) Range 4–21	Compared measures of problem behaviors and musicality	n/a (study 2 only for this area)
	(WS) 26 (PW) 16 (DS) 25	(WS) 20.88 (11.48) (PW) 19.38 (6.70) (DS) 18.83 (7.11) (All) Range 8–47	Compared measures of fears, anxieties, problem behaviors, and musicality	Greater range of emotional responses to music; WS reported experiencing both positive and negative emotions in response to negatively valenced music
Levitin et al., 2004	(WS) 118 (TD) 118 (ASD) 30 (DS) 40	(WS) 20.4 (10.4) Range: 5–50 (TD) 20.9 (7.4) Range: 5–44 (ASD) 18.2 (7.7) Range: 9–39 (DS) 17.2 (9.2) Range: 5–51	Salk McGill Music Inventory (SAMMI)^c^	High levels of emotional responsiveness; Emotional effects of music listening last longer in WS
Hopyan et al., 2001	(WS) 14 (TD) 14	(WS) 12 (3) (TD) 12 (3)	Affect identification task (auditory)	WS < TD CA identifying auditory affect;
Don et al., 1999	(WS) 19 (TD) 19	(WS) 8–13 (TD) 5–12	Parent Questionnaire/Interview	High levels of emotional responsiveness; Greater range of emotional responses to music

*ASD, Autism Spectrum Disorder; AVP, Arginine Vasopressin; CA, Chronological Age; DD, Developmentally Delayed Control; DS, Down Syndrome; EDA, Electrodermal Activity; HR, Heart Rate; OT, Oxytocin; PW, Prader-Willi Syndrome; TD, Typically Developing Control; WS, Williams Syndrome*.

a *Age in years, expressed in one of the following formats based on information available: (a) range in years, (b) mean (standard deviation), or (c) mean ± years*.

b *Musicality Interest Scale (MIS): Questionnaire with subscales in: (1) interest in and liking of music, (2) emotional reactions to music, and (3) musical skills*.

c *Salk McGill Music Inventory (SAMMI): Comprehensive questionnaire with subscales in: (1) demographic information, (2) interest in music, (3) emotional responsiveness to music, (4) music creativity and reproduction, (5) musical training, (6) age of onset of musical behavior*.

Of the studies that relied on parent report through various questionnaires or interviews (such as the MIS or SAMMI), the most frequently cited findings included an overall heightened emotional responsiveness to music, such as more intense and longer lasting emotional reactions, when compared to both TD peers and others with neurodevelopmental disorders (Don et al., 1999; Levitin et al., 2004). Other findings included a greater range of emotional responses to music (Don et al., 1999; Dykens et al., 2005); a significant correlation between emotional responsivity to music and social-emotionality (i.e., identification of another's emotions, desire to please, empathy, etc.) (Ng et al., 2013); and emotional responsiveness was most predicted by auditory sensitivities (Lense et al., 2013).

On tasks involving the identification of the emotional valence of various auditory or visual stimuli, individuals with WS tended to perform with mixed results. Some studies documented that individuals with WS performed comparatively to TD peers matched for CA (auditory: Bhatara et al., 2010; Järvinen et al., 2016; visual: Järvinen-Pasley et al., 2010; Lense et al., 2014b) while others reported poorer performance compared to TD (auditory: Hopyan et al., 2001; Järvinen et al., 2012; visual: Bhatara et al., 2010). However, individuals with WS tended to perform more accurately than individuals with ASD or other intellectual disabilities (Bhatara et al., 2010; Järvinen-Pasley et al., 2010). Within both the visual and auditory domain, WS exhibit a bias toward increased competence in identifying social (i.e., faces and voices) over non-social (i.e., images and music) affective stimuli (Järvinen-Pasley et al., 2010; Järvinen et al., 2012, 2016).

Lense et al. (2014b) examined the influence of a brief musical excerpt on the identification of visual affect. Although the emotional valence of the musical prime didn't have an effect on the accuracy in identifying the valence of the facial stimuli, individuals with WS demonstrated significantly faster reaction time when the valence of the target face matched the preceding music. Accuracy may have been impacted because the study used only two emotions (happy and sad) and the ease of identifying the emotions resulted in a ceiling effect, which was noted in the control group as well. Similarly, Järvinen-Pasley et al. (2010) showed a similar lack of improvement in accuracy for emotionally congruent over incongruent auditory/visual stimuli; however this could possibly be due to the exaggerated interest in faces in WS.

As expected, individuals with WS reported positive emotional states in response to positively valenced music. However, the same individuals reported personally experiencing both positive and negative emotional states in response to negatively valenced music (Dykens et al., 2005). This resonates with a consistent finding across studies that individuals with WS are more accurate in identifying the affective valence of positive (happy) over negative (sad or fear) stimuli (Hopyan et al., 2001; Järvinen et al., 2012, 2016) and rate the intensity of positive affect as more intense (Järvinen-Pasley et al., 2010). These findings suggest a possible lack of awareness or differentiation between more complex emotions such as fear, worry, sadness, and loneliness in individuals with WS, and also highlights the heterogeneity of their emotional responsiveness. Future studies are needed to examine reports of these more complex emotions and the perceptions or effects of more nuanced emotional states in music with individuals with WS.

A few studies examined responses of various autonomic systems to musical and auditory stimuli. Järvinen et al. (2012) found greater heart rate variability in response to vocal affect compared to musical affect, suggesting increased arousal to social auditory stimuli. Using a similar paradigm, Järvinen et al. (2016) found similar variability in heart rate along with significantly increased electrodermal activity in response to musical stimuli in the WS group, a pattern not found in the TD or ASD groups, suggesting further autonomic arousal in response to music. The WS and ASD groups also exhibited diminished habituation to both vocalizations and music over time, indicating both differential and sustained arousal compared to TD. In another autonomic area, Dai et al. (2012) found an increase in levels of oxytocin and arginine vasopressin in response to music in individuals with WS, despite elevated basal levels of these neuropeptides. However, these responses were also seen, although to a lesser extent for vasopressin, in response to a cold pressor test (placing a hand in ice cold water). Taken together, these findings suggest that the enhanced emotional responsiveness to music found in WS may be impacted by disruptions or dysregulation in autonomic reactivity.

The strengths of the articles included in this section are the inclusion of multiple types of control groups and the wide range of methodologies utilized. However, one limitation in comparing reports of emotional responsiveness in WS to others with neurodevelopmental disorders is the potential for responses to be impacted by the social and expressive nature of individuals with WS. Individuals with WS are more verbally expressive compared to others with neurodevelopmental disorders, which could lead parents of individuals with WS to be more aware of their internal experiences and reactions to music. It is also possible that some of the responses to music reported in this section are related to the presence of auditory stimuli in general, rather than specifically to the presence of music. Studies examining the role of auditory processing in emotional responses to music are needed.

Overall, the emotional responsiveness profile of WS in relation to music appears to present as heightened emotional responses to music, including more intense, longer lasting, and a wider range of emotional responses. These studies also suggest that differences in autonomic arousal in response to music or sound may account for some of these heightened responses. Future studies are needed to examine which brain areas are involved in these dysregulations, what qualities of music contribute to various emotional responses, and how music impacts social and emotional behavior in WS.

#### Musical processing: absolute pitch, amusia, and auditory processing

Four articles included findings related to various aspects of processing auditory stimuli (Table 5).

**Table 5:** Musical processing: absolute pitch, amusia, and auditory processing.

**Author/Year**	***N***	**Age^a^ (years)**	**Topic(s)**	**Task(s)**	**Finding(s)**
Lense et al., 2013	(WS) 73	(WS) 10–51	Amusia; Auditory Processing	Distorted Tunes Test (DTT)^b^; Spectral/Fundamental Processing Task (SPF)^c^	Higher incidence of amusia in WS compared to general population; Amusia strongly predicted musical skill; Overall tendency toward fundamental processing in WS
Martínez-Castilla et al., 2013 (2 studies)	(WS) 7 (TD) 14 (AP) 2	(WS Trained) 21.96 (6.8) Range: 15–32 (TD Trained) 21.49 (5.7) Range: unclear (AP) 14 and 16.4	AP	Pitch identification task: Label pitches without use of a reference tone	Prevalence of AP in WS is not higher than the general population; Both WS and TD performed equally and near chance
	(WS) 27 (TD) 54 (AP) 2	(WS Trained) 21.96 (6.8) (WS Untrained) 19.55 (5.94) (TD Trained) 21.49 (5.7) (TD Untrained) 19.59 (6.09) (AP) 14 and 16.4	AP	Pitch memory task: Discriminate if two tones were same/different following a retention interval filled with a distracting melody	
Deruelle et al., 2005	(WS) 16 (TD) 16	(WS) 12 y 7 m (4 y) Range: 8 y 7 m−19 y 3 m (TD) 13 y 5 m (3 y 7 m)	Global/Local	Discriminate if two melodies were same/different; “Different” melodies had errors that either violated or preserved the overall contour of the previous melody	Deficits in global rather than local perception of auditory stimuli in WS
Lenhoff et al., 2001	(WS) 5	(WS) 13–43	AP	Battery of tasks for absolute and relative pitch: identifying single notes; identifying natural notes in harmonic dyads/triads; pitch production and transposition	Higher prevalence of AP in WS than the general population; The critical period for acquisition of AP may be extended in WS

*AP, Absolute Pitch; TD, Typically Developing Control; WS, Williams Syndrome*.

a *Age in years, expressed in one of the following formats based on information available: (a) range in years, (b) mean (standard deviation), or (c) mean ± years*.

b *DTT, Distorted Tunes Test (Drayna et al., 2001)*.

c *SPF, Spectral/Fundamental Processing Task (Schneider et al., 2005; Wengenroth et al., 2010)*.

##### Absolute pitch

Absolute pitch (AP) is the rare ability to identify or produce the pitch of a sound without use of a reference tone. This particular skill occurs in 1 in 10,000 people, more commonly in cultures with tonal languages, and its acquisition is often associated with early musical exposure and training between the ages of 3–6 years (Takeuchi and Hulse, 1993).

Two of the included studies examined AP, presenting contradictory results. Lenhoff et al. (2001) tested five musically trained individuals with WS for abilities of AP. These individuals performed near ceiling levels on traditional measures for AP, leading authors to conclude that the prevalence of AP is higher among individuals with WS than the general population. Martínez-Castilla et al. (2013) calculated that, given the prevalence of WS in the US/Canada, this could indicate that the prevalence could be 10 times greater than TD populations. Lenhoff et al. also suggested that the critical period for developing AP may be extended in WS, given that all of the individuals in the study began their musical training after the proposed critical period for the general population.

The study of AP in individuals with WS presents some difficulty with respect to their cognitive limitations. Traditional measures of assessing AP require participants to be able to label pitches using traditional musical nomenclature (i.e., note names). Most musically trained individuals with WS do not learn music formally or learn how to read sheet music, making it difficult to conduct accurate assessments with a suitable sample size. Results from Lenhoff et al. (2001) may need to be interpreted with caution as the small sample of five participants all had musical training, were able to label musical notes, and therefore may not be representative of the larger WS population.

Martínez-Castilla et al. (2013) attempted to overcome this limitation by utilizing both traditional measures for AP and a novel paradigm examining participants' long-term memory for target stimuli. The latter does not require the ability to label musical notes and allowed for the study of a much larger sample, including participants with and without musical training. Performance on both measures was near chance for both the WS and control groups, contrasting the near ceiling level performance on both measures by two self-reported AP possessors. These results indicated that the prevalence of AP in WS is not remarkable. Additionally, performance on both measures was not associated with cognitive ability or musical training. Since all of the participants with WS began their musical training late and did not develop AP, Martínez-Castilla and colleagues refuted the findings of Lenhoff et al. (2001) related to the extended critical period for the development of AP.

Martínez-Castilla et al. (2013) pointed to minor differences between their study and that conducted by Lenhoff et al. (2001), including use of different pitch registers and differences in the number of items presented, however they argued these are unlikely to have had an effect on the results as modifications made were likely to make the task easier. Other possible explanations for the variation between the two studies could be explained by differences in musical training and cognitive level between the two samples. However, detailed information on musical training was not offered by Lenhoff et al. Also, although overall IQ was reported in both studies, Lenhoff et al. did not report their instrument used to assess cognitive functioning and the variability in cognitive functions in WS is widely known (see Martens et al., 2008, for review).

Some additional neuroanatomical evidence is also often cited when discussing AP. In TD populations, musicians with AP have shown a stronger leftward asymmetry in the planum temporale when compared to musicians without AP or non-musicians (Schlaug et al., 1995). This anatomical correlate was reported in individuals with WS by Hickok et al. (1995). However, subsequent reports have not replicated this finding (Galaburda and Bellugi, 2000) while others have found the opposite pattern (Eckert et al., 2006; Chiang et al., 2007).

The variability in behavioral and neuroanatomical findings within WS speaks to the need for continued inquiry. Future studies should continue to investigate AP using non-traditional tasks that do not require participants to label musical notes, such as discriminating whether pitches are the same or different (Martínez-Castilla et al., 2013) or using visual stimuli such as stairs to remember pitch intervals (Heaton et al., 2008). These studies should also examine left planum temporale volumes and their associations to performance on AP tests.

##### Amusia

On the opposite end of the spectrum, amusia is the inability to recognize or reproduce musical tones, despite typical cognitive abilities or exposure to music. Individuals with amusia have difficulty with basic music tasks such as recognizing and discriminating melodies, singing, distinguishing between meters, tapping along with a beat, and having poor musical memory (Sloboda et al., 2005). Amusia can be acquired through neurological damage; however congenital amusia is prevalent in 1.5–4% of the population (Kalmus and Fry, 1980; Peretz and Vuvan, 2017).

Lense et al. (2013) assessed a large sample (*n* = 73) of individuals with WS for characteristics of amusia using a battery of assessments including the Distorted Tunes Test (DTT; Drayna et al., 2001), a test for pitch amusia whereby participants heard a series of well-known songs, some of which were altered to have note errors (the contour and rhythm was preserved), and were asked if the song was played correctly. Eleven percent of participants met the criteria for amusia. Performance on the DTT was not associated with age, sensitivity to sound, family musical environment, and time spent playing/listening to music. It was moderately associated with IQ, number of types of musical training, cumulative duration of training, singing skill, and musical interest. DTT performance was negatively associated with a measure of spectral/fundamental processing (i.e., poorer DTT = generally mixed or somewhat spectral processing, see next section). Only musical training was a predictor of DTT performance and those who scored better on the DTT made fewer interval deviations, fewer contour errors, and sang at a slower tempo during a solo musical performance. This study was methodologically sound as it employed a battery of tests covering multiple areas of inquiry, allowing for comparison of multiple outcomes with a single large sample.

Although the DTT is the only outcome tool for amusia reported in this section, the Montreal Battery of Amusia (MBEA; Peretz et al., 2003) is another tool commonly used in the assessment of amusia. Lense et al. (2014a) argue that the DTT is a better task for individuals with WS given its highly engaging stimuli and the fact that the MBEA uses same/different tasks, which require working memory, which is impaired in WS. Future studies are needed to determine the most suitable assessment for amusia for individuals with WS and to examine the temporal qualities of music as they relate to amusia, as only findings related to pitch amusia have been reported.

##### Auditory processing (global/local and spectral/fundamental perception)

Musical sound perception is an incredibly complex phenomenon, with a high degree of individual variability. In the general population, there is an even distribution between those who perceive sound by decomposing it into its harmonics (spectral processing) and those who perceive sound based on the fundamental frequency (fundamental processing). Wengenroth et al. (2010) reported an extreme and nearly-uniform fundamental or holistic processing bias in individuals with WS, which is a marked deviation from the even distribution found in TD individuals in the control group.

Two studies (Lense et al., 2013; Lense and Dykens, 2016) have repeated this Spectral Fundamental Processing task (SPF; Schneider et al., 2005; Wengenroth et al., 2010). Lense et al. (2013) reported a range from extreme fundamental processing to somewhat spectral, with a mean of somewhat fundamental, in a sample of 73 individuals with WS. Also, all of the participants with WS identified as amusic within the study (*n* = 8) scored between the two processing styles, suggesting that a lack of a distinct processing style might contribute to perceptual deficits. Lense and Dykens (2016) did not report the results of the SPF collected in their study. However, beat and meter perception skills were predicted by a fundamental-processing style. Future studies are needed to examine the relationships between processing style and perceptual skills, specifically the influence of a fundamental processing style on rhythmic production skill.

Deruelle et al. (2005) examined tendencies for global and local perception of musical elements in children with WS by presenting pairs of melodies and asking participants if the two melodies were the same or slightly different. Some of the melodies in the global condition included a single pitch change that violated the overall contour of the first melody, representing a disruption in the global property of the melody. Melodies in the local condition included a similar pitch change, however this change was consistent with the overall contour of the melody and represented a change to the local properties of the melody. Individuals with WS performed similarly on both tasks; their performance was consistent with the control group for the local condition but far below the control group for the global condition. This deficit in global processing stands in contrast to the general advantage for global over local processing of musical stimuli in TD children (Ouimet et al., 2012) and is consistent with perceptual deficits for visuospatial stimuli in individuals with WS (Bihrle et al., 1989; Farran, 2005). These results have been replicated with adults with WS (Elsabbagh et al., 2010).

Although the studies in this section reported findings in different areas of auditory and musical perception, a consistent theme across these studies is the pervasiveness of atypical auditory processing in individuals with WS. Although these divergent auditory processes may present with a high level of variability within WS, the pervasiveness with which they are reported indicates a need for future research to help explain how differences in auditory processing impact the way individuals with WS interact with music.

#### Brain imaging and morphology

Five of the included articles employed various neuroimaging tests (Table 6), including magnetic resonance imaging (MRI), functional magnetic resonance imaging (fMRI), diffusor tensor imaging (DTI), and electroencephalogram (EEG).

**Table 6:** Brain imaging and morphology.

**Author/Year**	***N***	**Age^a^ (years)**	**Imaging**	**Areas examined**	**Findings(s)**
Lense et al., 2014a	(WS) 17	(WS) 16–48	MRI DTI	Primary auditory cortex (STG, TTG); Pars orbitalis of IFG and SLF	Decreased connectivity along the superior longitudinal fasciculus
Lense et al., 2014b	(WS) 13 (TD) 13	(WS) 27.1 ± 7.1 (TD) 27.7 ± 6.0	EEG	n/a	Increased evoked alpha in response to happy vs. sad; Increased evoked gamma in response to congruent affective stimuli
Martens et al., 2010	(WS) 25 (TD) 25	(WS) 8–41 (TD) 8–41	MRI	Primary auditory cortex Planum temporale	Larger bilateral planum temporale, no difference in asymmetry
Thornton-Wells et al., 2010	(WS) 13 (TD) 13	(WS) 16–33 (TD) 17–27	MRI fMRI	Not stated, appears to be entire brain, no a priori	Activation of occipital and early visual areas in response to music
Levitin et al., 2003	(WS) 5 (TD) 5	(WS) 28.8 (14.6)	fMRI	STG, MFG, SFG, cerebellum, amygdala, cingulate gyrus, pons	More diffuse activation; Decreased temporal lobe activation; Increased right amygdala activation

*DTI, Diffusion Tensor Imaging; EEG, Electroencephalogram; fMRI, Functional Magnetic Resonance Imaging; IFG, inferior frontal gyrus; MFG, medial frontal gyrus; MRI, Magnetic Resonance Imaging; SFG, superior frontal gyrus; SLF, superior longitudinal fasciculus; STG, superior temporal gyrus; TD, Typically Developing Control; TTG, transverse temporal gyrus; WS, Williams Syndrome*.

a *Age in years, expressed in one of the following formats based on information available: (a) range in years, (b) mean (standard deviation), or (c) mean ± years*.

Using structural MRI, Martens et al. (2010) found similar planum temporale and primary auditory cortex volumes between individuals with WS and TD controls, despite significantly smaller overall brain volume in the WS group. In other words, bilateral planum temporale volumes were proportionally larger in individuals with WS. Although no overall significant difference in asymmetry was found, left planum temporale volume was greater in a subset of WS participants who performed better on various musical production tasks (SAT; Nickson and Black, 1962) and was positively correlated with the ability to sing the final note of a melodic phrase. Although larger, the difference in primary auditory cortex volume was not significant.

Lense et al. (2014a) examined neural correlates of amusia in a sample of individuals with WS, some of whom met the criteria for amusia. Diffusion tensor imaging (DTI) revealed that individuals with amusia displayed decreased connectivity along the superior longitudinal fasciculus (SLF), a fiber pathway that connects the temporal and frontal lobes. This remained significant after controlling for musical training, suggesting that amusia is not a result of a lack of musical experiences. Lense et al. (2014a) confirmed that these findings are highly consistent with previous research on amusia in TD populations and suggest amusia is related to poor connectivity rather than dysfunction in the primary auditory cortex in both populations.

Two fMRI studies examined individuals with WS while undergoing various listening tasks. Levitin et al. (2003) examined auditory and emotional areas in a small sample of individuals with WS and TD controls while they listened to various excerpts of classical music, noise, and silence. Results revealed remarkable differences between the two groups, indicating more variable and widely diffuse activations in WS participants in contrast to the well-defined activations of TD controls. Individuals with WS demonstrated significantly reduced activation in the temporal lobes (superior temporal gyrus, medial temporal gyrus, superior temporal sulcus), whereby all of these areas were found to be areas of activation for TD controls. Significant bilateral temporal lobe activation was evident for both groups, although to a lesser degree for the WS group, while listening to noise, demonstrating the ability to distinguish between music and noise processing on a neuroanatomic level. A notable finding in the WS group was significantly greater activation in the right amygdala, suggesting a possible neural correlate for the heightened emotional reactions to music in individuals with WS. Other findings included consistent activation of the cerebellum, pons, and brainstem in the WS group.

In a similar task, Thornton-Wells et al. (2010) found 19 significant clusters of activation that were different between the WS and TD groups. Similar increased activations as Levitin et al. (2003) were found in the cerebellum and bilateral emotional areas (insula, parahippocampal gyrus, posterior cingulate). Amygdala activation was found, however it did not reach significance. In contrast, increased activations in the bilateral superior temporal gyrus were also found. However, findings revealed activation of occipital and early visual areas in response to music and other auditory stimuli, most notably to simple notes and chords. These findings were also observed in two follow-up studies using subsets of participants, although not in all participants and not in all conditions. This inconsistent activation of visual areas in response to auditory stimuli is different than classic synesthesia, in which cross-modal processing is reliable across conditions.

Lense et al. (2014b) measured EEG oscillatory activity during an affective priming task, whereby participants heard brief emotionally valenced musical excerpts and were asked to make judgments about the emotional valence of facial stimuli. Individuals with WS demonstrated greater evoked alpha power in response to happy musical excerpts than compared to sad, which reflects sensory processing and attentional control. This activity was positively associated with parental ratings of emotional reactions to music on the Musical Interest Scale (MIS; Blomberg et al., 2006). Individuals with WS also demonstrated significantly greater evoked gamma activation in response to facial stimuli that matched the valence of the musical prime, which is believed to be associated with integration across sensory modalities.

Conducting neuroimaging tests such as fMRI present unique challenges given the prevalence of anxiety and hearing sensitivities in individuals with WS. Both Levitin et al. (2003) and Thornton-Wells et al. (2010) sought to address this by orienting their WS participants to the fMRI prior to the procedure. This process involved sending participants audio and video recordings of the fMRI machine to introduce them to the sounds of the machine, use of an fMRI simulator, interacting with the machine and staff prior to the scan, and speaking to an individual with WS who had previously completed multiple scans. Such supports may be beneficial in future neuroimaging studies.

At face value, many of these findings suggest a neurological basis for the unique relationship with music found in individuals with WS, including the effects of more diffuse neurological activation, activation of emotional centers, and cross-modal activation of auditory and visual/affective areas in response to music. At a fundamental level, these studies also provide further support for the pervasiveness of differential or atypical processing of auditory information in individuals with WS.

Limitations of articles included in this section speak to the need for controls with other developmental disabilities (e.g., DS). Comparison to these groups may help to clarify the weight of atypical auditory processing on the affinity for music found in this population as other populations may have similar differences in processing auditory information but lack the characteristic affinity found in WS. Also, given the small number of studies included in this section, the need for replication using various methods is evident as the studies in this section present little converging results. Given the pervasiveness of atypical auditory processing in individuals with WS, future neuroimaging studies would benefit from a broader consideration of a priori regions to include regions involved in the entire network of auditory processing, which may explain in part the affinity for music in WS.

#### Cognitive processes: memory and math

Three of the included articles examined the relationships between music and various cognitive processes (Table 7).

**Table 7:** Cognitive processes: memory and math.

**Author/Year**	***N***	**Age^a^ (years)**	**Topic(s)**	**Task(s)**	**Finding(s)**
Dunning et al., 2015	(WS) 44	(WS) 8–48	Verbal memory	Recall a list of sentences that were either spoken or sung to a novel melody	WS with musical training demonstrated improved verbal memory on both sung and spoken conditions
Martens et al., 2011 (2 studies)	(WS) 38	(WS) 6–59	Verbal memory	Recall a list of sentences that were either spoken or sung to a familiar melody	WS with musical training demonstrated improved verbal memory when sentences were sung > spoken
	(WS) 38	(WS) 7–50			
Reis et al., 2003	(WS) 16	Unclear; Only DOB given	Math Skills	10-day music and math curriculum focused on understanding fractions	Majority of participants increased their understanding of math concepts; Expressed increased confidence in math

*WS, Williams Syndrome*.

a *Age in years, expressed in one of the following formats based on information available: (a) range in years, (b) mean (standard deviation), or (c) mean ±years*.

##### Memory

Two of the included articles examined the effects of melody on word learning as a facet of verbal memory. Martens et al. (2011) presented two sets of participants with WS with a memory task requiring them to recall a list of sentences that were either spoken or sung to a familiar melody. Participants with prior musical training, in the form of private lessons, demonstrated significantly better long-term recall of the sentences when they were sung compared to when they were spoken. This was not observed for participants without musical training and was not correlated to musical enjoyment, time spent listening to music as a child, or heightened emotional reactions to music. A follow-up study examining both short and long-term verbal memory found identical results for long-term recall and no effect on short-term recall. Authors also noted that participants exhibited greater attention and were more still during conditions in which the sentences were sung, regardless of musical training.

Dunning et al. (2015) found nearly identical results when the sentences were sung to a novel melody. In this study, those with formal musical training performed significantly better with sung or spoken sentences than those without musical training. Although not significant, participants without musical training also performed better when the information was presented through song. Performance on the memory task was not impacted by age, verbal IQ, musical enjoyment, length of time listening to music per day, or length of participation in music lessons, choir, or music therapy.

##### Math

As part of the *Music* and *Minds* program, a 10-day intensive music program for individuals with WS, Reis et al. (2003) reported a positive influence of music on acquisition of math skills. Participants attended two daily math sessions focused on proficiency with fractions, including practical applications to time, money, measurement, musical notes, and objects. This program incorporated music as both a learning tool and instructional methodology throughout the duration of the program. Authors reported that a majority of the participants increased their understanding of the covered math concepts and expressed increased confidence related to fractions following the program. Unfortunately, details on the instructional and assessment methods used were not reported.

Although available literature in this section is sparse, together these findings indicate a potential for clinical intervention addressing cognitive processes using music. Both studies examining memory provide converging results that transmitting information melodically can improve long-term recall for those with musical training. Also, anecdotal reports of increased attention during musical tasks and confidence following a musical program speak to the merit of the inclusion of music to support the acquisition of cognitive skills in WS. However, future studies should seek to substantiate these claims and further explore protocol in employing musical strategies to effectively enhance acquisition of cognitive skills in WS.

#### Fears, anxieties, and problem behaviors

Three of the included articles examined relationships between music and fears, anxieties, and problem behaviors in individuals with WS (Table 8), which are known to be phenotypic characteristics of the population. Blomberg et al. (2006) collected questionnaire data from two separate familiar respondents of 38 subjects with WS. Many significant correlations were found between measures of hyperacusis and fears. Data collected on fears using the Fear Survey Schedule for Children-Revised (FSSC-R; Ollendick, 1983) revealed high levels of fears, which was consistent in level and types of fears with previous research (Dykens, 2003). Thirteen percent of the participants scored above the suggested cutoff for hyperacusis, which is five times greater than the percentage found in the general population using the same outcome measure [Hyperacusis Questionnaire (HQ); (Khalfa et al., 2002)]. However this is much lower than previous reports of prevalence of hyperacusis in WS (Klein et al., 1990; Gothelf et al., 2006) and consistent with studies using narrower definitions of hyperacusis (Levitin et al., 2005).

**Table 8:** Fears, anxieties, and problem behaviors.

**Author/Year**	***N***	**Age^a^ (years)**	**Topic(s)**	**Task(s)**	**Finding(s)**
Lense and Dykens, 2013b (study #2 only)	(WS) 13	(WS) 7–49	Fears	Measured salivary cortisol before and after a prepared solo music performance in front of mixed familiar/unfamiliar audience	No significant change in cortisol in response to musical performance; Baseline cortisol significantly correlated with rated musical skill
Blomberg et al., 2006	(WS) 38	(WS) 10–50	Fears	Compared measures of fears, hyperacusis, and musicality	Fears and anxieties could be associated with hyperacusis
Dykens et al., 2005 (2 studies)	(WS) 31 (PW) 26 (DS) 32	(All) 4–21	Problem behaviors	Compared measures of problem behaviors and musicality	Externalizing symptoms negatively correlated with listening to music; Internalizing symptoms negatively correlated with producing music;
	(WS) 26 (PW) 16 (DS) 25	(All) 8–47	Fears Anxiety Problem behaviors	Compared measures of fears, anxieties, problem behaviors, and musicality	Lower levels of fear and anxiety associated with increased frequency, skill, and duration in producing music

*DS, Down Syndrome; PW, Prader-Willi Syndrome; WS, Williams Syndrome*.

a *Age in years, expressed in one of the following formats based on information available: (a) range in years, (b) mean (standard deviation), or (c) mean ±years*.

Blomberg and colleagues further noted few associations between musicality and hyperacusis or fears. A weak significant negative correlation was found between musical skill and the “failure and criticism” subscale of the FSSC-R, indicating that musically-accomplished individuals with WS are slightly more resilient to perceptions of criticism. A weak significant positive correlation was found between emotional reactions to music and measures of hyperacusis. This finding is contrary to the early hypothesis concerning the protective factors of musicality in preventing or managing anxiety. However, it is also consistent with findings from Lense et al. (2013) that emotional responsiveness was predicted by auditory sensitivities in individuals with WS.

Using similar measures, Dykens et al. (2005) found that lower levels of fears and anxiety were associated with increased frequency, duration, and skill in producing music. Results using the Child Behavior Checklist (CBCL; Achenbach, 1991) indicated that externalizing symptoms (such as aggressive behaviors or non-compliance) were negatively correlated with frequency of listening to music and internalizing symptoms (such as anxiety, depression, withdrawal, somatic complaints, etc.) were negatively correlated with producing music in individuals with WS. This suggests that, independent of musical skill, producing or listening to music may have positive effects on maladaptive behaviors and anxiety.

Lense and Dykens (2013b) examined cortisol reactivity during a solo musical performance in individuals with WS as a measure of psychological stress. Increases in cortisol are often seen in TD populations during times of social stress and evaluation, such as public performances or presentations (Beck et al., 2000; Taylor et al., 2010). Cortisol measures, taken prior to and 20 min following a prepared solo musical performance in front of a live audience, remained stable, and showed no significant change. Although this could be interpreted as a lack of psychosocial stress related to a musical performance, it is also possible that the participants in the study could have experienced significant anticipatory anxiety in preparation for the performance and thus exhibited higher baseline cortisol, which could account for the lack of reactivity following the performance. Baseline cortisol was found to be strongly associated with musical skill, as rated by judges during the performance, but not with music listening or anxiety. As it is hard to draw conclusions from a lack of an identified effect, future studies are needed to clarify this finding using more frequent measures of cortisol surrounding musical performance.

Overall, two of these studies (Dykens et al., 2005; Blomberg et al., 2006) share similar limitations in that they are reliant on parent report, which poses possible bias and the risk for underestimation related to mental health information. The results of these studies are also correlational and do not indicate if these associations are the product or cause of fears and anxiety. Finally, it is worth noting that fear and anxiety are not the same, as fear is more of an immediate response and anxiety is a more future-oriented process. The role of music in mitigating the future-oriented aspects of anxiety as compared to the immediate experience of fear may be different. Given the known prevalence of anxiety and fear in this population (Dykens, 2003) and the limited number of studies in this area, future research is needed to examine the potential impact of facets of musicality and musical skill on overall levels of fear and problem behaviors, which may indicate an avenue for clinical intervention.

#### Methodological critique: a critical review

Of the 31 articles included, all but one that included more than one study either utilized entirely new samples or added new participants to a subset of participants from the first sample. For this reason, when analyzing various methodological aspects of the included articles it was determined to interpret these results through the number of studies as opposed to the number of articles, as the samples for these studies were largely different.

##### Method of diagnosis

Over the past 30 years, there have been many developments in methods for diagnosing WS. This was reflected in the variability of reporting the diagnostic methods in the included studies. Early cases of WS relied on the medical and clinical phenotype to receive a diagnosis of WS. Since the 1990s, DNA testing using *fluorescent in situ hybridization* (FISH) has become the most widely used test of genetic confirmation for WS (Lowery et al., 1995). Of the included studies, 56% reported their participants' diagnoses were genetically confirmed using the FISH test; 31% reported diagnoses were “genetically confirmed” but did not specify the method; 11% reported the use of a phenotype index (Preus, 1984; Pérez-Jurado, 1997; American Academy of Pediatrics, Committee on Genetics, 2001); 8% were unclear, as diagnostic information was either included in a demographics questionnaire but not reported, reported for some participants and unknown for others, or reported the age of diagnosis but not the method; and 14% did not mention confirmation of diagnosis. Many of these studies also reported that their participants exhibited characteristics of the clinical phenotype in addition to their reported methods of diagnosis. Although there has not been any indication that having confirmatory genetic testing has a significant impact on the findings of previous studies, the scientific rigor of WS research would be enhanced by emphasizing the importance of utilizing a genetic and phenotypic confirmation of WS when recruiting participants for future studies (Martens et al., 2008).

##### Method of recruitment

The low prevalence of WS presents a challenge when recruiting participants. Of the included studies: 31% recruited participants from a music camp; 25% recruited from a convention or conference; 31% recruited from a national WS association (from the USA, Canada, Spain, or Sweden); 22% recruited from an established research program or genetics clinic; 8% recruited from a parent support group; and 6% were either unclear or did not report their method of recruitment. Many of these studies recruited from more than one source.

##### Matching of control groups

The appropriate method for matching TD control groups has been the subject of debate. Individuals with WS have been compared to TD controls based on CA as well as those of similar MA or cognitive level. Only two studies (6%) (Levitin and Bellugi, 1998; Don et al., 1999) matched for MA, both of which were published before the year 2000. Since then, the remainder of studies utilizing TD controls (61%) matched for CA. Twenty-two percent matched for sex/gender and 11% matched for musical training. Although it was possible to infer based on available information, 19% of the studies did not explicitly state their matching criteria. One study utilized participants with WS as a control group and thus does not fit into any of the above categories.

##### Types of control groups

Various types of control groups were recruited for comparison purposes throughout the included studies: 64% TD control groups; 8% ASD; 11% DS; and 6% Prader-Willi Syndrome (PW). Some of the studies included multiple control groups. Nearly one-third of the studies (31%) did not utilize a control group, including only participants with WS. Future studies should consider including both TD controls and controls with other developmental disabilities or cognitive deficits in order to better characterize the diverse range of musicality in individuals with WS.

##### Reporting IQ

Nine different tests of cognitive function were utilized across the included studies. Given the time-span covered in this study, some of these were revised versions of the same test. Overall, 39% of the studies used the Kaufman Brief Intelligence Test and 33% used various versions of the Wechsler Intelligence Scale. Some of the studies, particularly those with samples of a wide age range, employed multiple measures depending on the age of the participants. Fourteen percent of the studies reported IQ-values but did not list which test was used and 6% reported using a test but did not include the resulting values. Fourteen percent did not assess for IQ.

##### Reporting hearing loss and sensitivities

Screening for both hearing loss and sensitivities for participants with WS is particularly relevant to studies of music given that musical stimuli fall under the auditory domain and particularly in light of the increased prevalence of high frequency hearing loss in this population (Cherniske et al., 2004; Gothelf et al., 2006). Of the included studies: 44% did not screen for hearing loss or sensitivities; 31% relied on parent report but did not specify the method of collection; 17% relied on parent report through a mentioned questionnaire (8% Sensitivities to Sounds Questionnaire, Lense and Dykens, 2013a; 6% SAMMI, Levitin et al., 2004; 3% HQ, Khalfa et al., 2002); and 8% utilized a threshold audiometry test.

##### Testing limitations

Future studies may benefit from consideration of methodological challenges in evaluating individuals with WS:

- Discrimination tasks involving determinations of “same vs. different” require verbal memory, a skill that may be impaired in individuals with WS (Don et al., 1999). Hopyan et al. (2001) utilized three different pre-tests to confirm understanding of the concepts of same/different. Future studies may benefit from such screening measures or the assessment of verbal memory as a covariate.
- Several studies reported near ceiling effects for TD participants (Järvinen-Pasley et al., 2010; Martens et al., 2010; Martínez-Castilla and Sotillo, 2014). The modifications to tasks that may be required to accurately evaluate individuals with WS may make tasks too easy for TD, limiting the comparisons made between groups.
- Many studies utilized perceptual ratings by trained judges (Musicality: Lense and Dykens, 2013a,b; Lense et al., 2013; Tonal/Rhythm: Levitin and Bellugi, 1998; Martínez-Castilla and Sotillo, 2008; Martínez-Castilla et al., 2011). Although these judges were trained and inter-rater reliability was established, these perceptual judgments are subject to bias and may be impacted by each judge's musical background. Martínez-Castilla and Sotillo (2008) found that acoustical analysis of a musical performance produced more accurate and reliable results than ratings by either musicians or non-musicians.
- When utilizing multiple test measures, consideration should be given to task length, complexity, and order. Don et al. (1999) and Hopyan et al. (2001) both assessed pitch and rhythm discrimination skills. Participants in both of these studies scored worse on the rhythm test, which always followed the pitch test. Thus, test order was a possible confound for these studies. Don et al. also reported attentional difficulties during testing, which could have differentially affected performance on these tasks.
- Similarly, 23% of the included studies reported excluding participants based on the inability to understand or follow directions, inability to attend to task, refusal to participate in structured task, or presence of recording artifacts during neuroimaging. Future studies may benefit from improved screening measures to assess for these issues.
- Considering the disposition toward social interaction among individuals with WS, presentation of live vs. recorded musical stimuli may impact results as individuals with WS may interact differently with socially presented stimuli.
- Other known methodological issues include small sample sizes [excluding survey data from Levitin et al. (2004), the mean WS sample size among the included studies was 23], large age ranges in samples, methods of matching controls, and potential bias related to methods of recruitment.

##### Proposed reporting guidelines

Considering the methodological and reporting limitations outlined above, future studies should:

- Assess and report the following, including instruments used and results:
  - IQ
  - Hearing loss and sensitivities
  - Musical training (both duration and types)
- Additionally identify and report:
  - Method of diagnosis or genetic confirmation
  - Method of recruitment of participants
  - Note whether stimuli are presented via live or recorded audio.

### Risk of biases

Two common biases were found throughout the included studies that are known biases of many studies of individuals with WS. The first of these is related to the recruitment of participants. Nearly half (47%) of the included studies recruited participants from either a summer music camp or a national convention. Both of these methods are predisposed toward those who attend such events and are potentially not representative of the general WS population. This may be of particular relevance in the study of the relationship between music and WS. Although previous involvement in music or heightened musical skill are generally not requirements to attend such camps, individuals who attend summer music camps may be predisposed toward greater musical involvement or interest.

The second bias is related to the reliance on parent report. Roughly 75% of the included studies utilized parent report for various outcome tools, questionnaires, and to report hearing sensitivities and musical training. Although the demographic information gathered related to diagnosis and musical training leaves little room for error, relying on parent report to determine prevalence of hyperacusis, levels of anxiety, emotional responsiveness to music, musical interest, or engagement, musical skill, etc. leaves room for under or over reporting of this information. However, Fisher et al. (2014) attest to the accuracy of parent-report over self-report with individuals with WS. Thus, use of parent report may be the most accurate method of assessment in some cases.

## Discussion

This review identified 31 articles examining the relationship between WS and music. These articles were divided into seven categories, many of which align with general phenotypic characteristics of the syndrome.

### Summary of main findings

#### Williams syndrome and music: perspective on the phenotype

Overall, the musical profile of individuals with WS appears to be deeply rooted in their musicality. This is expressed through consistent reports of heightened interest in music, a greater propensity toward musical activities, and heightened emotional responsiveness to music. Although many individuals with WS share a strong attraction to music, a smaller percentage demonstrate strong musical skills. Results found in this review point to a high degree of variability in skill and engagement in music, also suggesting that overall musicality may not predict musical skill. Individuals with WS appear to present with *relatively* good musical skills that are more in line with their cognitive abilities than CA. Musical strengths for this population seem to be based more in expressivity and musicality over formal musical skills.

Considering the difficulty and subjectivity inherent in operationalizing the concept of “musicality,” the authors recommend reporting findings related to musicality under four major headings so that all facets can be studied, but reported in a manner that better facilitates comparison with other findings: Affinity (e.g., interest, preferences, enjoyment, motivation); Experience [e.g., musical training, community involvement (i.e., band, choir, church, etc.)]; Engagement (e.g., time spent playing/listening, attending concerts); and Artistry (e.g., creativity, expressivity, sensitivity, emotionality). Other definitions may include functions of musical skill, but the authors recommend reporting these results separately as the above headings more accurately represent the musical phenotype of WS, apart from musical skill. Future studies would benefit from operationalizing the individual aspects of musicality under these headings to examine their role in the WS phenotype more closely.

Atypical auditory processing, autonomic irregularities, and differential neurobiology might underlie this affinity for music and other aspects of the phenotype. It is unlikely that the deletion of genetic material found in individuals with WS directly predisposes these same individuals toward a greater interest in music. Instead, it is highly plausible that the resulting differences in structural and functional anatomy uniquely affect the manner in which music is perceived and processed within this population. However, it is important to remember that the phenotype in neurodevelopmental disorders does not fully emerge from the outset, but develops gradually over time (Martínez-Castilla et al., 2016). Thus, it is difficult to tease apart the biological and sociological influences that relate to this unique relationship between WS and music.

#### Music and IQ

When examining the effects of cognitive ability (IQ) on performance during musical tasks, multiple studies identified a correlation between task performance and measures of IQ (Martínez-Castilla et al., 2011, 2016; Lense and Dykens, 2016). This is also supported by the numerous reports of WS performance falling below TD peers matched for CA, yet fairly similar to TD peers matched for MA. However, other studies found no such relationships (Hopyan et al., 2001; Martínez-Castilla and Sotillo, 2008, 2014). Considering these contradictory findings in light of the atypical cognitive profile of WS, it is possible that performance on these tasks is more predicted by specific cognitive processes than an overall measure of intellectual ability. Future studies should continue to examine specific cognitive skills and cognitive developmental milestones as potential covariates when comparing performance on tasks to TD peers.

#### Perceptual abilities

The “creative completions” noted by Levitin and Bellugi (1998) may explain some of the variance in skill for perceptual or discrimination tasks reported in this review and also points to an interesting disparity in the perceptual abilities of individuals with WS. On a task requiring participants to accurately reproduce a given rhythm by clapping, errors produced by participants with WS tended to be more rhythmically compatible with the target rhythm than the comparison group, thus preserving the overall structure of the original rhythm. Given this, it may also be true that on tasks requiring participants to judge whether two rhythms are the same or different, participants with WS may judge two items as being the same if they share similar overall rhythmic structures instead of comparing them on the basis of individual beats (Hopyan et al., 2001). Similarly, acoustical analysis of song-singing revealed that individuals with WS make many more errors in accuracy for individual pitches than those that alter the overall contour of the melodic phrase (Martínez-Castilla and Sotillo, 2008). In summary, individuals with WS seem to conserve the overall structure, contour, or idea of a musical phrase better than they can discriminate or reproduce it exactly.

On one hand, individuals with WS may be better at conserving global musical structures, such as melody and meter, than their component parts, such as individual pitches, beats, or rhythms. Yet, on the other hand, this stands in contrast to visuospatial deficits that have been reported in individuals with WS, which show remarkable deficits in the processing of global over local aspects of visual stimuli (Bihrle et al., 1989). In the auditory domain, Deruelle et al. (2005) reported similar deficits in the perception of global contour in melody compared to local pitch elements. Possible explanations for this striking disparity could be the inherent difference between production and discrimination tasks or it may be that these tasks may require different cognitive skills. Basic pattern perception skills are stronger in individuals with WS than auditory rote-learning or working memory, which are necessary for various same/different discrimination tasks (Don et al., 1999; Martínez-Castilla et al., 2016). Future studies should continue to examine this relationship and should consider examining perceptual skills in both the auditory and visual domains within the same sample.

The high prevalence of pitch amusia in WS stands in contrast to their reported attraction to music, however this can be seen as congruent with the lack of preserved musical skill. This may also indicate that the attraction to music may fall more heavily on non-pitch related aspects of music (e.g., rhythm, dynamics, timber, etc.).

#### Clinical considerations

Many articles proposed the potential for including music in both clinical and educational interventions with individuals with WS. However, to date, no published studies have examined the clinical use of music with this population. Articles point to the heightened interest in and motivation for musical activity (Don et al., 1999; Levitin et al., 2004) as well as greater attention during musical vs. non-musical activity (Martens et al., 2011) within the WS population as rationale for incorporating music into clinical intervention to assist in modulating and maintaining arousal, attention, and engagement with clinical tasks. Proposed target domains of intervention among the included studies were diverse, covering: social and communication skills (Lense and Dykens, 2016); language and prosody skills (Martínez-Castilla and Sotillo, 2014); auditory-motor connections (Lense and Dykens, 2013a); emotional understanding and sensitivity (Ng et al., 2013); management of anxiety (Dykens et al., 2005); attention and concentration (Lense and Dykens, 2013a); and educational outcomes (Martens et al., 2011; Dunning et al., 2015).

Existing research on the effects of music on non-musical function is promising for many of the above areas. Active use of instruments in synchrony with a musical beat supports optimal kinematics through the coupling of auditory and motor processes (Thaut, 2005) and the provision of temporal limits that cue and constrain movement (Thaut et al., 1991; Lim et al., 2011). Playing musical instruments also provides the opportunity to practice fine and gross motor skills, which may be a more preferred and motivating activity for many individuals with WS. Pairing novel information to music has been shown to be an effective tool for enhancing recall of target information (Gfeller, 1983; Wolfe and Horn, 1993; Knott, 2017). Furthermore, the structure and predictability provided by the rhythmic aspects of music supports and guides attention (Thaut, 2005; Geist and Geist, 2012). Considering the proposed potential and existing clinical literature outside of WS, future studies are needed to explore musical intervention with individuals with WS.

Although unpublished literature was not included within the scope of this study, multiple theses and dissertations were identified during the formal search process that have begun to examine the effects of music on non-musical function in individuals with WS. Appendix C in Supplementary Material lists these identified unpublished theses and dissertations. However, this list may not be exhaustive as unpublished literature was not within the scope of this study.

## Conclusions

The affinity for music observed in this population since the earliest of case reports has been given considerable attention. This review contributes to the existing literature by examining the unique relationship between WS and music through a systematic and comprehensive research lens. The knowledge gained from this review provides guidance for future researchers to more fully understand the relationship between music and individuals with WS and to explore how music can be used to provide therapeutic interventions.

## Author contributions

DT developed search procedure and materials, performed database search, reviewed articles for inclusion/exclusion, extracted data from articles, analyzed extracted data, wrote manuscript, and revised manuscript for submission. MM provided guidance related to developing search materials, analyzing extracted data, and writing the manuscript; revised, and edited manuscript for submission. DS is thesis committee chair and edited final manuscript. ER provided comments on the manuscript.

### Conflict of interest statement

The authors declare that the research was conducted in the absence of any commercial or financial relationships that could be construed as a potential conflict of interest.
