# Supplementary material for: Williams Syndrome and Music: A Systematic Integrative Review
Source: Front Psychol. 2018 Nov 14;9:2203. doi: 10.3389/fpsyg.2018.02203 (PMC6246687; doi:10.3389/fpsyg.2018.02203)
Supplement: Supplementary file 1 [file Data_Sheet_1.docx]

Supplementary Material

Williams syndrome and music: A systematic integrative review

Donovon Thakur^*^, Marilee Martens, David Smith, Ed Roth

*** Correspondence:** Donovon Thakur, Donovon.C.Thakur@wmich.edu

# Supplementary Figures and Tables

See below

**Appendix A: Exclusions Based on Full-Text and Reasons for Exclusion**

| **Author(s)** | **Year** | **Title** | **Publication** | **Reason for Exclusion** |
| --- | --- | --- | --- | --- |
| Carrico, A. H. | 2015 | Constructing a two-way street: An argument for interdisciplinary collaboration through an ethnomusicological examination of music therapy, medical ethnomusicology, and Williams syndrome | *Voices: A World Forum for Music Therapy, 15(3), 1-16* | Positional Paper |
| Carrico, A. H. | 2014 | Discovering "diffability": Musical experiences and perspectives of individuals with Williams syndrome at Whispering Trails | Thesis:  Florida State University | Unpublished Thesis |
| Don, A. J. | 1999 | Auditory pattern perception in children with Williams syndrome | Dissertation:  University of Windsor | Unpublished Dissertation |
| Elsabbagh, M.  Cohen, H.  Karmiloff-Smith, A. | 2010 | Discovering structure in auditory input: Evidence from Williams syndrome | *American Journal on Intellectual and Developmental Disabilities, 115(2), 128-139* | Music not focus of article;  Auditory Perception |
| Fidler, D. J.  Lawson, J. E.  Hodapp, R. M. | 2003 | What do parents want?: An analysis of education-related comments made by parents of children with different genetic syndromes | *Journal of Intellectual and Developmental Disability, 28(2), 196-204* | Music minimal focus of article |
| Heaton, P.  Allen, R. | 2009 | "With concord of sweet sounds...": new perspectives on the diversity of musical experience in autism and other neurodevelopmental conditions | *Annals of the New York Academy of Sciences, 1169, 318-325* | WS minimal focus of article;  Book chapter,  published in journal |
| Kwak, E. E. | 2009 | An exploratory study of the use of music therapy in teaching mathematical skills to individuals with Williams Syndrome | Dissertation:  Michigan State University | Unpublished Dissertation |
| Lenhoff, H. M. | 2009 | Soaking in the music | *Exceptional Parent, 39(3), 40-45* | Magazine Article |
| Lenhoff, H. M. | 1998 | Information sharing: Insights into the musical potential of cognitively impaired people diagnosed with Williams syndrome | *Music Therapy Perspectives, 16(1), 33-36* | Informational Article |
| Lenhoff, H. M.  Wang, P. P.  Greenberg, F.  Bellugi, U. | 1997 | Williams syndrome and the brain | *Scientific American, 277(6), 68-73* | Magazine Article |

**Appendix A cont.: Exclusions Based on Full-Text and Reasons for Exclusion** **cont.**

| **Author(s)** | **Year** | **Title** | **Publication** | **Reason for Exclusion** |
| --- | --- | --- | --- | --- |
| Lense, M. D.  Dykens, E. M. | 2011 | Musical interests and abilities in individuals with developmental disabilities | *International Review of Research in Developmental Disabilities, 41, 265-312* | Book Series/  Review |
| Levitin, D. J. | 2005 | Musical behavior in a neurogenetic developmental disorder: Evidence from Williams Syndrome | *Annals of the New York Academy of Sciences, 1060, 325-334* | Review Article |
| Mackenzie, L. B. | 2005 | ‘When words fail, music speaks’: How communication transforms identity through performance at the Berkshire Hills Music Academy | Dissertation:  University of Massachusetts Amherst | Music not focus of article;  Unpublished Dissertation |
| Maher, B. A. | 2001 | Music, the brain, and Williams syndrome: Rare disorder offers insight into the genetic basis of cognition | *Scientist, 15(23), 20-21* | Partially Music  Magazine Article |
| Milne, H. | 2004 | The development of talent in young adults with Williams syndrome: An exploratory study of ecological influences | *Australasian Journal of Special Education, 28(2), 79-101* | Data re-published in  Reis et al. (2003) |
| Milne, H. | 2002 | A comparative case study of persons with Williams syndrome and musical interests | Dissertation:  University of Connecticut | Unpublished Dissertation |
| Pridmore, M. D. | 2014 | Affective priming effect of music on emotional prosody in Williams syndrome | Thesis:  Middle Tennessee State University | Unpublished Thesis |
| Stambaugh, L. | 1996 | Special learners with special abilities | *Music Educators Journal, 83(3), 19-23* | Music minimal focus of article;  Magazine Article |
| Sacks, O. | 1995 | Musical ability | *Science, 268(5211), 621-622* | Letter |
| Wengenroth, M.  Blatow, M.  Bendszus, M.  Schneider, P. | 2010 | Individual MRI segmentation reveals enlarged auditory cortex in Williams syndrome as a neural substrate of training-independent musicality | *Child Neuroradiology, 20(3), 196-197* | Conference Abstract/  Unable to Retrieve |
| Wengenroth, M.  Blatow, M.  Bendszus, M.  Schneider, P. | 2010 | Leftward lateralization of auditory cortex underlies holistic sound perception in Williams syndrome | *PLoS One, 5(8), 1-10* | Not Music;  Auditory Perception |

**Appendix B: Data Extraction Form**

| **Thesis ID**  *1-3 authors:*  *Author, Author, & Author (yyyy)*  *4+ authors:*  *Author et al. (yyyy)* |  |
| --- | --- |
| **Report ID #** *(from endnote)* |  |
| **Name/ID of Individual**  **Performing Data Extraction** |  |
| **Date Form Completed** *(dd/mm/yyyy)* |  |

1. **General Information**

| 1. **Author(s)** |  |
| --- | --- |
| 1. **Year** |  |
| 1. **Title of Publication** |  |
| 1. **Journal** *(journal, volume(issue), pages)* |  |
| 1. **Keywords** |  |
| 1. **Database(s) accessed** |  |
| **Notes:** | |

1. **Eligibility**

| **Study**  **Characteristics** | **Inclusion Criteria** *(exclude if no)* | **Yes/No/Unclear** | **Location in text**  *(pg & ¶/fig/table)* |
| --- | --- | --- | --- |
| 1. **Date Range** | Published before 2017 (through Dec 2016) |  |  |
| 1. **Subject(s)  of study** | Williams syndrome |  |  |
|  | Music |  |  |
| 1. **Language** | English |  |  |
| 1. **Publication** | Published in a peer-reviewed journal |  |  |
| **Study**  **Characteristics** | **Exclusion Criteria** *(exclude if yes)* | **Yes/No/Unclear** | **Location in text**  *(pg & ¶/fig/table)* |
| 1. **Participants** | Participants include a mixture of individuals with WS and individuals with other diagnoses and outcomes are not differentiated by the authors |  |  |
|  | Participants have comorbid diagnoses of WS and another diagnosis |  |  |
| 1. **Subject(s)  of study** | Hyperacusis without reference to music |  |  |
| 1. **Design of study** | Review/Magazine/Positional/etc. |  |  |
| 1. **Decision:** |  | | |
| 1. **Reason for exclusion** |  | | |
| Notes:  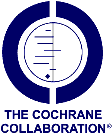  *Form modified from* Data Extraction Form 2013 08 12 available from Cochrane Website.  *Effective Practice and Organization of Care (EPOC). Data collection form. EPOC Resources*  *for review authors. Oslo: Norwegian Knowledge Centre for the Health Services; 2013.*  *Available at: http://epoc.cochrane.org/epoc-specific-resources-review-authors* | | | |

**DO NOT PROCEED IF STUDY EXCLUDED FROM REVIEW**

Notes on using a data extraction form:

- Be consistent in the order and style you use to describe the information for each included study.

Record any missing information as unclear, not described, or N/A, to make it clear that the information was not found in the study report(s), not that you forgot to extract it

1. **Methods**

|  | **Descriptions as stated in report/paper** | **Location in text**  *(pg & ¶/fig/table)* |
| --- | --- | --- |
| 1. **Aim of study** |  |  |
| 1. **# of Reports** *(if more than one)* |  |  |
| Notes: | | |

1. **Participants and Groups**

*Label each group within each box (where applicable), using consistent labeling for each row.*

*Copy and paste this table for multiple reports/experiments within the same article.*

| **Participants** | **Description as stated in report/paper** | **Location in text**  (pg & ¶/fig/table) |
| --- | --- | --- |
| 1. **Total N** |  |  |
| 1. **Group name(s)** |  |  |
| 1. **Number in group (n)** |  |  |
| 1. **Control Group(s) matched by:** |  |  |
| 1. **Age** *(range, mean, etc.)* |  |  |
| 1. **Sex** *(M = n; F = n)* |  |  |
| 1. **Race/Ethnicity** |  |  |
| 1. **Method of Diagnosis** |  |  |
| 1. **Method(s) of recruitment of participants** |  |  |
| 1. **Inclusion/Exclusion criteria** *(used in study)* |  |  |
| 1. **IQ Values** |  |  |
| **IQ Method of Collection** |  |  |
| 1. **Hearing Screening** |  |  |
| **Hearing Screening  Method of Collection** |  |  |
| 1. **Musical Training** |  |  |
| **Musical Training  Method of Collection** |  |  |
| 1. **Other Screening** *(aside from IQ/Hearing)* |  |  |
| 1. **Other treatment(s) received** *(in addition to study intervention)* |  |  |
| Notes: | | |

1. **Tasks and Outcomes**

*Number each outcome within each box, using consistent numbering for each row.*

*Copy and paste this table for multiple reports/experiments within the same article.*

|  | **Description as stated in report/paper** | **Location in text**  *(pg & ¶/fig/table)* |
| --- | --- | --- |
| 1. **Description of task(s)**   *(general description of each task or tool)* |  |  |
| 1. **Setting**   *(including location and social context)* |  |  |
| 1. **Outcome name(s)** |  |  |
| 1. **Outcome tool(s)** |  |  |
| 1. **Gathered from**   *Participants, parent report, etc.* |  |  |
| 1. **Outcome definition(s)**   *(operational definition)* |  |  |
| Notes: | | |

1. **Results**

*List each outcome appropriately under Quantitative/Qualitative*

*Copy and paste table for multiple reports/experiments within the same study.*

| 1. **For Quantitative Data** | **Description as stated in report/paper** | **Location in text**  *(pg & ¶/fig/table)* |
| --- | --- | --- |
| **Outcome** |  |  |
| **Outcome** |  |  |
| **Outcome** |  |  |
| Notes: | | |

1. **Other information**

|  | **Description as stated in report/paper** | **Location in text**  *(pg & ¶/fig/table)* |
| --- | --- | --- |
| 1. **Key conclusions of study authors** |  |  |
| 1. **Suggested areas for further research** |  |  |
| 1. **References to other relevant studies** |  |  |
| Notes: | | |

1. **Risk of Bias assessment**

| **Domain** | **Risk of bias**  *Low/High/Unclear* | **Support for judgment** | **Location in text**  *(pg & ¶/fig/table)* |
| --- | --- | --- | --- |
| 1. **Sampling bias**   *(self-selection: predisposition toward involvement)* |  |  |  |
| 1. **Self/Parent Report biases**   *(reporting on behalf of another; social desirability bias)* |  |  |  |
| 1. **Other bias** |  |  |  |
| Notes: | | | |

1. **Applicability**

| 1. **Does the study directly relate to the review question?** | *Yes/No/Unclear* | *Any issues of partial or indirect applicability?:* |
| --- | --- | --- |
| 1. **Which area(s) of the review does this publication relate to?** |  | |
| Notes: | | |

**Appendix C: Unpublished Theses and Dissertations**

| **Author** | **Year** | **Title** | **Type/University** |
| --- | --- | --- | --- |
| Carrico, A. H. | 2014 | *Discovering "diffability": Musical experiences and perspectives of individuals with Williams syndrome at Whispering Trails* | Thesis  Florida State University |
| Pridmore, M. D. | 2014 | *Affective priming effect of music on emotional prosody in Williams syndrome* | Thesis  Middle Tennessee State University |
| Barnett, B. A. | 2013 | *Does novel music improve verbal memory in individuals with Williams syndrome?* | Thesis  The Ohio State University |
| Ross, G. | 2011 | *Perception of emotional expression in musical performance by individuals with Williams syndrome* | Thesis  Western Michigan University |
| Woitulewicz, L. M. | 2011 | *The effects of background music, rhythm, and noise on a sustained attention task in adults with Williams syndrome* | Thesis  Western Michigan University |
| Kwak, E. E. | 2009 | *An exploratory study of the use of music therapy in teaching mathematical skills to individuals with Williams Syndrome* | Dissertation  Michigan State University |
| Hata, M. | 2006 | *A survey of music therapists regarding the efficacy of music therapy in the treatment of children and adolescents with Williams syndrome* | Thesis  Western Michigan University |
| Mackenzie, L. B. | 2005 | *‘When words fail, music speaks’: How communication transforms identity through performance at the Berkshire Hills Music Academy* | Dissertation  University of Massachusetts Amherst |
| Milne, H. | 2002 | *A comparative case study of persons with Williams syndrome and musical interests* | Thesis  University of Connecticut |
| Don, A. J. | 1999 | *Auditory pattern perception in children with Williams syndrome* | Dissertation  University of Windsor |
| Pawuk, L. G. | 1999 | *A comparison of rhythmical abilities and behaviors between typical children and children with Williams Syndrome* | Thesis  Western Michigan University |
